# The Superior Colliculus: Cell Types, Connectivity, and Behavior

**DOI:** 10.1007/s12264-022-00858-1

**Published:** 2022-04-28

**Authors:** Xue Liu, Hongren Huang, Terrance P. Snutch, Peng Cao, Liping Wang, Feng Wang

**Affiliations:** 1 Shenzhen Key Lab of Neuropsychiatric Modulation, Guangdong Provincial Key Laboratory of Brain Connectome and Behavior, CAS Key Laboratory of Brain Connectome and Manipulation, the Brain Cognition and Brain Disease Institute, Shenzhen Institute of Advanced Technology, Chinese Academy of Sciences, Shenzhen-Hong Kong Institute of Brain Science-Shenzhen Fundamental Research Institutions, Shenzhen, 518055 China; 2 University of Chinese Academy of Sciences, Beijing, 100049 China; 3 Michael Smith Laboratories and Djavad Mowafaghian Centre for Brain Health, University of British Columbia, Vancouver, V6T 1Z4 Canada; 4 National Institute of Biological Sciences, Beijing, 100049 China

**Keywords:** Superior colliculus, Glutamatergic neurons, GABAergic neurons, Neuronal circuits, Innate behaviors

## Abstract

The superior colliculus (SC), one of the most well-characterized midbrain sensorimotor structures where visual, auditory, and somatosensory information are integrated to initiate motor commands, is highly conserved across vertebrate evolution. Moreover, cell-type-specific SC neurons integrate afferent signals within local networks to generate defined output related to innate and cognitive behaviors. This review focuses on the recent progress in understanding of phenotypic diversity amongst SC neurons and their intrinsic circuits and long-projection targets. We further describe relevant neural circuits and specific cell types in relation to behavioral outputs and cognitive functions. The systematic delineation of SC organization, cell types, and neural connections is further put into context across species as these depend upon laminar architecture. Moreover, we focus on SC neural circuitry involving saccadic eye movement, and cognitive and innate behaviors. Overall, the review provides insight into SC functioning and represents a basis for further understanding of the pathology associated with SC dysfunction.

## Introduction

The superior colliculus (SC), and the homologous optic tectum (OT), are highly conserved midbrain structures in vertebrates [1–4], which play a critical role in integrating multi-modal signals to assess saliency and promote action. Depending upon the species, the SC is a laminar structure with seven or eight layers. The mammalian SC is often considered in terms of its organization of three distinct groups of layers. Based on neuronal distribution patterns, connectivity, and functional properties, the alternating layers of neurons and fibers in the SC of all vertebrates can be roughly subdivided into either the superficial (SCs), intermediate (SCi), and deep layers (SCd) in mammals, or in the OT of non-mammals, into the superficial, central, and periventricular layers [5–10]. The SC receives topographically-organized sensory information and forms cognitive maps related to multiple behaviors. The SCs, organized into the stratum zonale, griseum superficiale, and opticum, receives input from retinal ganglion cells (RGC) and the striate visual cortex, forming a topographical localization unit responding to visual information [6, 11–13]. Unlike the SCs, neurons in the SCi and SCd receive inputs from somatosensory and auditory sources in addition to those from the basal ganglia and cerebellum, and have larger receptive fields that respond to visual information related to orienting movements and specific behaviors [14]. Notably however, much of our knowledge of the SC remains at the level of these layers and sub-regions rather than neuronal cell types and circuits associated with specific behaviors.

The SC primarily contains gamma-aminobutyric acid (GABA)ergic [7, 15–18] and glutamatergic neurons [19]. Although the SC engages numerous afferent and efferent inputs from the retina [20], cortex, and subcortex [21–23], findings to date have demonstrated only specific connections as having certain laminar- and cell-type-specific characteristics [19]. GABAergic neurons in the SC receive inputs from RGCs [24] and the brainstem parabrachial region [25], and project to the ventral tegmental area (VTA) [24]. Glutamatergic SC neurons receive inputs from the ventral posteromedial thalamic nucleus [22], substantia nigra pars reticulata (SNr) [26], ipsilateral pretectal nuclear complex [27], and cortex [22], and project to the VTA [28], zona incerta (ZI) [29], central mesencephalic reticular formation [30], lateral posterior thalamus [31], and the periaqueductal grey [32].

Over the past decade, there has been a surge of interest in the SC due to its critical roles in visual information processing [1, 33], sensorimotor integration [34], visual orientation, and cognitive functions [3, 35–37], including selective visual attention [35, 38] and decision making [18, 35, 39]. However, our understanding of the neural mechanisms involving the SC which bring about these functions remains relatively rudimentary. With advancing technological developments, it is becoming possible to answer long-standing questions regarding the anatomical organization of the SC, the specific neuronal types and circuits underlying behavior, and to shed light on critical comparative and evolutionary questions. For example, glutamatergic SC neurons and their component circuits are thought to play a crucial role in vision-evoked innate fear and hunting behavior [29]. GABAergic SC neurons and their afferent and efferent connections are thought to play a critical role in wakefulness [24] and eye movements [17]. This suggests that cell-type-specific circuit-based behavioral heterogeneity is a reflection of SC neuronal activity which has diverse phenotypic characteristics combined with distinct circuitry and structural brain connections.

To accelerate the identification of neurons projecting onto SC cellular subpopulations, several groups have taken advantage of recent methodological advances in viral trans-synaptic tracing. In addition, a variety of approaches in transgenic rodents have revealed that discrete SC neuronal phenotypes and single neural-circuit elements may confer distinct roles, including eye movement [30, 40–42], cognitive behaviors [39, 43], visually-evoked innate fear [28, 32, 44–46], prey capture [29, 47, 48], sleep [24, 49], and drinking behavior [50]. These studies reinforce the hypothesis that different SC functions are mediated by diverse subpopulations of SC neurons organized in distinct neuronal networks. In this review, we describe our current understanding of the organization of the SC, including the molecular and physiological diversity of neuronal populations, local circuits, and long-range connections with a focus on anatomical sub-regions, specific cell types, and evolutionary conservation across vertebrates. Moreover, we also discuss recent evidence for specific neural circuits in the regulation of innate behavior across species and highlight the involvement of the SC in neurodegenerative and neuropsychiatric disorders.

## Neuronal Subtypes in the SC

Recent studies indicate that different afferent/efferent SC networks, which generate complex and distinct functional SC circuits, are correlated with distinct neuronal phenotypes [19, 24, 28, 29]. The SC consists of several neuronal subtypes, which have diverse expression of transcription factors, cell adhesion molecules, neuropeptides, Ca^2+^-binding proteins, and receptors [45, 51–53] (Table 1). Future work should focus on patterns of cell-type-specific synaptic connections in the major types of SC neurons to understand the circuit mechanisms underlying sensorimotor processing and transformation. As pieces of this connectivity puzzle fall gradually into place, the knowledge obtained can guide efforts to understand structure–function relationships in SC circuits.

**Table 1:** Molecular and physiological diversity of superior colliculus/optic tectum neurons.

Transcription factor	Cell adhesion	Neuropeptide/Ca^2+^-binding protein	Neurotransmitter	Receptor
Rorβ	Cadherin 7	Parvalbumin	GABA	GABA_A_Rs, GABA_B_Rs, GABA_C_Rs
Brn3b	Netrin G2	Somatostatin	Glutamate	AMPA, NMDA receptor, mGluRs
ETV1	Protocadherin 20	Vasoactive intestinal polypeptide	Noradrenaline	Adrenergic receptor
Pitx2	Contactin 3	Corticotropin-releasing factor	Acetylcholine	Nicotinic acetylcholine receptor
NF-κB	Cadherin 6	Calbindin, Calretinin	Serotonin	5-HT1A and 5-HT1B receptors
Pax7	ALCAM	Substance P	Dopamine	D1 receptor, D2 receptor
Fork head-5		Gastrin releases peptides		neuropeptide Y receptor type 2
Cbln2		Otx2		Neurotensin receptor 1
		Cholecystokinin		Cannabinoid receptor 1

### GABAergic Neurons

GABA is synthesized from glutamate by glutamic acid decarboxylase 1 (GAD1, also known as GAD67) or GAD2 (also known as GAD65) and packed into vesicles by the vesicular GABA transporter (VGAT, also known as VAAT). Using the detection of GAD or VGAT as an index, it has been shown that a large population of GABAergic neurons [15, 51, 54–56] is present throughout the SC in many species including rats, mice, cats, rabbits, opossums, tree shrews, and rhesus and cynomolgous monkeys [17]. The locations and properties of these cells likely play critical roles in the regulation of SC functions. Moreover, in cats, GABAergic neurons account for 45% of the total SCs cell population and 30% of SCi neurons [7], whereas in mice, ~30% of SCs neurons are GABAergic [15, 17]. These GABAergic neurons exhibit a variety of morphologies, physiological properties, synaptic inputs and outputs, and are found both in the presence or absence of the Ca [2]^+^-binding protein parvalbumin (PV) [15, 51], vasoactive intestinal peptide [57], somatostatin, and cholecystokinin [58]. It is known that GABAergic SC neurons are essential for acute dark induction of wakefulness [24] and decision-making [18]. However, little is known of the subpopulations of inhibitory neurons and their laminar distribution. The full array of functions associated with GABAergic neurons in different SC laminae has still to be elucidated.

### Glutamatergic Neurons

Glutamatergic neurons can be identified by the expression of vesicular glutamate transporter 2 (VGLUT2) or in transgenic mice in which reporter fluorescent proteins are expressed under regulation of the *vGlut2* promoter. There are a variety of glutamatergic SC projection neurons in rodents [1, 26, 29, 59] and tree shrews [60]. In cats, there are similar amounts of tecto-tectal glutamatergic and GABAergic neurons in the SCi and SCd layers [61]. Moreover, recent studies have shown that glutamatergic projection neurons in the SCs [19] and SCd [32] encode distinct sensorimotor transformations for hunting and defensive behavior, respectively, implying that glutamatergic SC neurons exhibit heterogeneity which includes the marker genes they express, their laminar location, the circuit in which they are integrated, and their physiological function.

The identification of subpopulations of in SC subregions in vertebrates based on single-cell sequencing is still in its infancy [19]. Further investigation is required to clarify the elaborate cell-type diversity of GABAergic and glutamatergic neurons in different laminar SC regions and to determine the functions associated with these neuronal sub-types.

## Molecular and Physiological Diversity in the SC/Optic Tectum Neurons (Table 1)

Phenotypic identification of cell types enables us to better understand cellular heterogeneity and function. Along with rapid single-cell sequencing and other high-throughput approaches, identification of SC neuronal subtypes is becoming increasingly refined. Recent studies of the SC have used immunohistochemistry, *in situ* hybridization, and single-cell transcriptomic analyses to reveal more than 40 molecules related to SC function. These include: (1) transcription factors, such as retinoid-related orphan receptor β, brain-specific homeobox/POU domain protein 3β, Ets variant gene 1 [62], Pitx2 [63], nuclear factor kappa-B [64], Pax7 [65], fork head-5 [66], and Cbln2 [19]; (2) cell adhesion molecules, such as cadherin 7, contactin 3, netrin G2, cadherin 6, protocadherin 20, corticotropin-releasing factor 1 [62], and activated leukocyte cell adhesion molecule [67]; (3) neuropeptides/Ca^2+^-binding proteins, such as substance P, somatostatin, vasoactive intestinal peptide, PV, gastrin-releasing peptides, vesicular glutamate transporter 2, calbindin calretinin [62], and Otx2 [68]; and (4) neurotransmitters, including noradrenaline [69] acetylcholine [25], serotonin [53], dopamine [70], GABA [52], and glutamate [71], and receptors, including NMDA receptors, GABA_A_ [27, 72, 73], GABA_B_ [54, 74] and GABA_c_ [75, 76], adrenergic receptors [69], metabotropic glutamate receptor [77], nicotinic acetylcholine receptor, dopamine D1 and D2 receptors [70], 5-hydroxytryptamine (5-HT) 1A and 5-HT1B receptors, neuropeptide Y receptor type 2 [78], neurotensin receptor [79] and cannabinoid 1 receptor [80].

These differentially-expressed molecules serve as valuable markers for examining the molecular mechanisms that regulate the development and phenotype of the different SC neuronal subtypes, as well as marking certain behaviors. For example, nuclear factor kappa-B in the SC modulates processes following visual-system damage, including regeneration and visual system short-term processes [64] and activated leukocyte cell adhesion molecule in the SC is important for mediolateral axon targeting in the formation of retino-collicular maps [67]. Other examples include Pitx2 neurons in the SCi, which drive three-dimensional head movements during foraging behavior [63] and activation of PV^+^ excitatory neurons in the SC, which trigger innate fear responses [45]. Looking ahead, more comprehensive gene expression profiles such as spatial transcriptomics and *in situ* sequencing studies [81, 82] are expected to uncover sophisticated neuronal–behavioral classifications in the SC. Moreover, high-throughput DNA sequencing mapping of entire circuits with single-neuron precision [83] and spatially resolved transcriptomics [84] holds unmatched promise in unraveling the organization of brain cell types and their connectivity, circuit dynamics, and their relationship to behavior and disease.

## Basic SC Anatomy, Lamination, and Connectivity

The laminar structure of the SC includes the SCs, SCi, and SCd, which have distinct projections with corresponding functions. Identifying the afferent and efferent connections of each SC layer provides important clues regarding the functions mediated by each layer [35]. For instance, discovering why squirrels, tree shrews, and some arboreal primates have the most distinct lamination within the SCs layer would benefit our knowledge of visual processing. Recent methodological advances, including the use of adeno-associated virus (AAV), rabies virus, herpes simplex virus, and AAV-Retro-Cre combined with AAV-DIO-mCherry for monosynaptic and/or polysynaptic neuronal network tracing, have also facilitated the mapping of brain structures that project to the SC as well as projections from specific SC layers to defined brain structures. Moreover, cell-type-specific transgenic mouse lines provide the possibility of identifying SC neuronal subtypes. It is possible to selectively manipulate cell types within these mouse lines through the use of optogenetics and/or designer receptors exclusively activated by designer drugs. Detailed cross-species comparisons using modern molecular methods for identifying cell-type-specific SC afferents and efferents will be a productive area for investigations of SC evolution, lamination, neuronal cell types, and synaptic connectivity.

### Overview of Axonal Afferents onto SC Neurons (Summarized in Figs. 1 and 2)

**Fig. 1:**
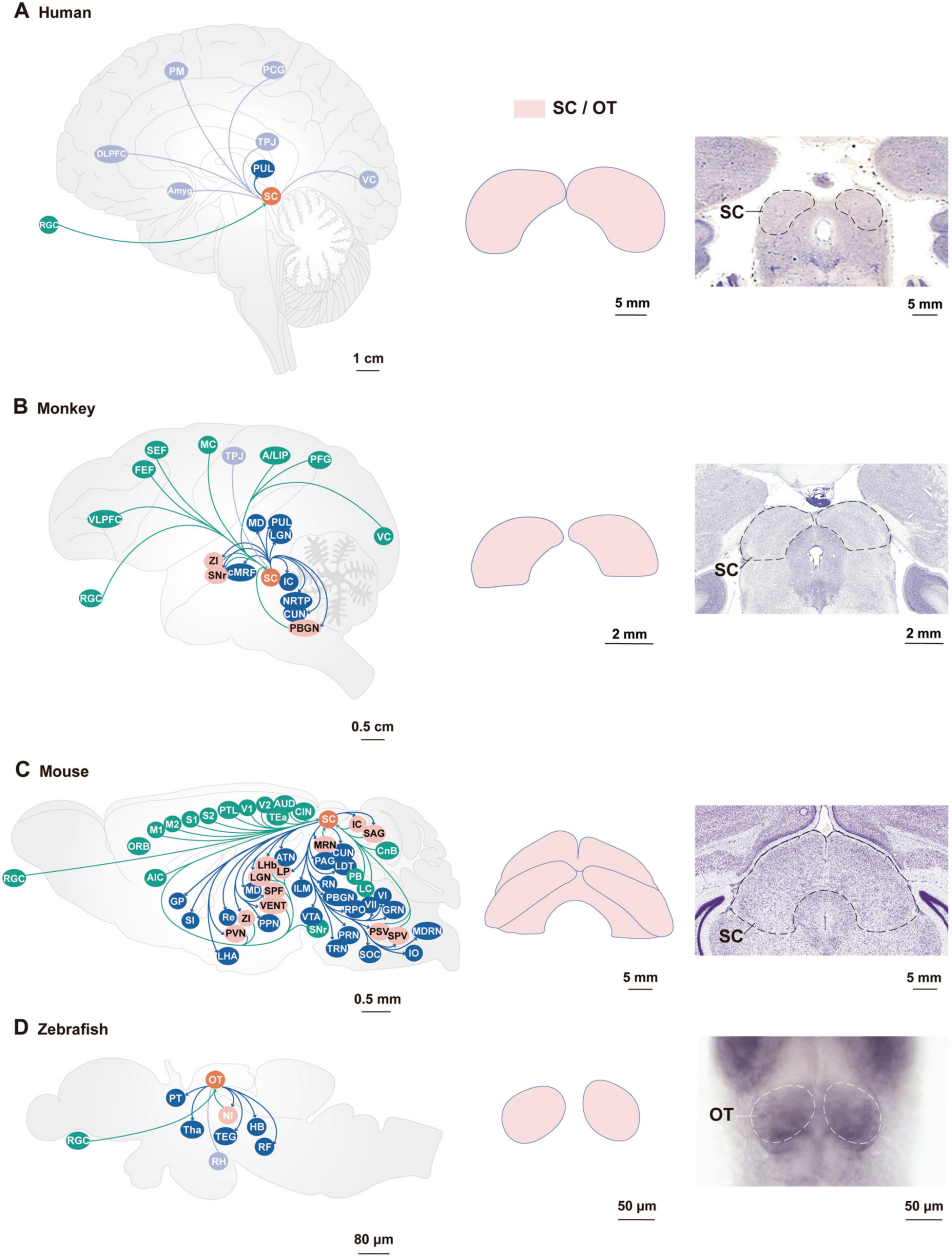
Superior colliculus/optic tectum afferent and efferent connections across vertebrates. **A** Humans. **B** Rhesus monkeys. **C** Mice. **D** Zebrafish. Using schematic sagittal brain sections, the figure shows homologous circuits across species for the superior colliculus (SC) and optic tectum (OT), with inputs to the SC in green and SC outputs in blue. Light blue represents SC connectivity, which is not yet fully determined. Light red indicates that the nucleus is reciprocally connected with the SC/OT. The middle and right-hand columns show schematics and coronal histological sections, respectively, of the SC or OT in different species. AIC, agranular insular cortex; A/LIP, anterior/lateral intraparietal area; Amyg, amygdala; AUD, auditory cortex; ATN, anterior group of the dorsal thalamus; CIN, cingulate cortex; CnB, cerebellar nucleus; cMRF, central mesencephalic reticular formation; CUN, cuneiform nucleus; DLPFC, dorsolateral prefrontal cortex; FEF, frontal eye field; GP, globus pallidus; GRN, gigantocellular reticular nucleus; HB, hind brain; IC, inferior colliculus; ILM, intralaminar nuclei of the thalamus; IO, inferior olivary complex; LHA, lateroanterior hypothalamic nucleus; LC, locus coeruleus; LDT, laterodorsal tegmentum; LGN, lateral geniculate nucleus; LHb, lateral habenular; LP, lateral posterior thalamic nucleus; M1, primary motor cortex; M2, secondary motor cortex; MC, motor cortex; MD, mediodorsal thalamus; MDRN, medullary reticular nucleus; MRN, midbrain reticular nucleus; MT, middle temporal area; NI, nucleus isthmi; NRTP, nucleus reticularis tegmenti pontis; ORB, orbital area; PAG, periaqueductal grey; PB, parabrachial nucleus; PBGN, parabigeminal nucleus; PCG, postcentral gyrus; PFG, inferior parietal lobule area 7b; PUL, pulvinar; PM, premotor cortex; PN, pretectal nucleus including anterior, medial, and posterior; PT, pretectum; PTL, parietal association cortex; PPN, pedunculotegmental nucleus; PRN, pontine reticular nucleus; PSV, principal sensory nucleus of the trigeminal; PVN, paraventricular nucleus of the hypothalamus; Re, reuniens thalamic nucleus; RF, reticular formation; RGC, retinal ganglion cell; RH, rostral hypothalamus; RN, raphe nucleus; RPO, nucleus raphe pontis; S1, primary somatosensory cortex; S2, secondary somatosensory cortex; SAG, nucleus sagulum; SEF, supplementary eye field; SI, substantia innominata; SNr, substantia nigra pars reticulata; SOC, superior olivary complex; SPF, subparafascicular nucleus; SPV, spinal nucleus of the trigeminal; TEa, temporal association area; TEG, tegmentum; Tha, thalamus; TPJ, temporoparietal junction; TRN, tegmental reticular nucleus; V1, primary visual cortex; V2, secondary visual cortex; VC, visual cortex; VENT, ventral group of the dorsal thalamus; VI, abducens nucleus; VII, facial motor nucleus; VLPFC, ventral lateral prefrontal cortex; VTA, ventral tegmental area; ZI, zona incerta. Right panel of **A** is adapted from Michigan State University, https://brains.anatomy.msu.edu/brains/human/coronal/2390_cell_labelled.html. Right panels of **B** and **C** are from the Allen Institute for Brain Science http://www.blueprintnhpatlas.org/static/referencedata, http://mouse.brain-map.org/static/atlas, Right panel of **D** from The Zebrafish Information Network http://zfin.org/ZDB-IMAGE-011218-26.

**Fig. 2:**
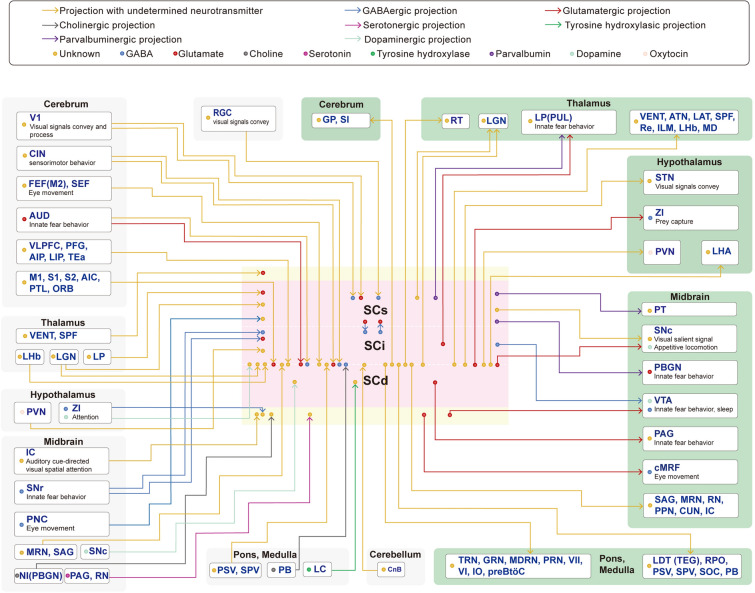
Inputs to and outputs from superior colliculus/optic tectum neurons. Schematic of the synaptic connections identified to date. Superior colliculus (SC) GABAergic neurons receive inputs from retinal ganglion cells (RGC), the primary visual cortex (V1), cingulate cortex (CIN), auditory cortex (AUD), GABAergic inputs from the midbrain substantia nigra pars reticulata (SNr), and cholinergic inputs from the brainstem parabrachial region (PB). Glutamatergic SC neurons receive inputs from GABAergic neurons from the SNr, glutamate inputs from the AUD, and projections with undetermined neurotransmitters from the CIN, V1, primary motor cortex (M1), primary somatosensory cortex (S1), secondary somatosensory cortex (S2), parietal association cortex (PTL), agranular insular cortex (AIC), orbital area (ORB), lateral posterior thalamus (LP), ventral group of the dorsal thalamus (VENT) including ventral medial and posteromedial nuclei of the thalamus, and the subparafascicular nucleus (SPF). SC neurons also receive inputs from the cerebral cortex that mainly involve the ventrolateral prefrontal area (VLPFC), inferior parietal lobule area 7b (PFG), anterior and lateral intraparietal area (AIP and LIP), temporal association area (TEa), retrosplenial area cortex (RSP), prelimbic area cortex (PL), infralimbic area cortex (ILA), secondary motor cortex (M2) including dorsal and ventral premotor cortex, frontal eye field (FEF), and the supplementary eye field (SEF). Moreover, SC neurons receive thalamic input from the lateral habenula (LHb), lateral geniculate complex (LGN), midbrain inputs from the inferior colliculus (IC), midbrain reticular nucleus (MRN), and nucleus sagulum (SAG), and hindbrain input from the principal sensory nucleus of the trigeminal (PSV), spinal nucleus of the trigeminal (SPV), and the cerebellar nuclei (CnB). Also, SC neurons receive inputs from GABAergic and dopaminergic projections from the zona incerta (ZI), oxytocin-positive neurons in the paraventricular nucleus of the hypothalamus (PVN), midbrain GABAergic inputs from the pretectal nuclear complex (PNC), cholinergic inputs from the nucleus isthmi (NI), dopaminergic inputs from the substantia nigra pars compacta (SNc), serotonergic inputs from the periaqueductal grey (PAG) and raphe nucleus (RN), and a hindbrain tyrosine hydroxylase projection from the locus coeruleus (LC). SC glutamate neurons target the LP and PAG, GABA neurons of the ventral tegmental area (VTA), ZI and central mesencephalic reticular formation (cMRF). SC GABA neurons target VTA dopamine neurons. SC parvalbumin-positive neurons target the parabigeminal nucleus (PBGN), pretectum (PT) and the LP. SC neurons also target the globus pallidus (GP) and substantia innominata (SI), thalamic nuclei including the thalamic reticular nucleus (RT), LGN, pulvinar (PUL) and VENT, anterior group (ANT), lateral group (LAT) of the dorsal thalamus and reuniens thalamic nucleus (Re), as well as the SPF, intralaminar nuclei of the thalamus (ILM), LHb, mediodorsal thalamus (MD), the hypothalamus area including the subthalamic nucleus (STN), PVN, lateral hypothalamic area (LHA) and midbrain nucleus SNc, SAG, MRN, red nucleus (RN), pedunculopontine nucleus (PPN), CUN, inferior colliculus (IC) and hindbrain area tegmental reticular nucleus (TRN), gigantocellular reticular nucleus (GRN), medullary reticular nucleus (MDRN), pontine reticular nucleus (PRN), facial nucleus (FN), abducens nucleus (VI), nucleus raphe pontis (RPO), PSV, SPV, superior olivary complex (SOC), PB, laterodorsal tegmental nucleus (LDT) and its homologous structure the tegmentum (TEG), and inferior olivary complex (IO), and the pre-Bötzinger complex (preBötC). Lines and arrows extending into the box for the SCs, SCi, and SCd indicate that these pathways have confirmed subregion targeting within the SC. Lines and arrows outside the box indicate that targeting of these pathways within the SC has not been shown to have subregion specificity.

#### SC Inputs in Humans and Non-human Primates

The primate SC conveys visual information from the retina [85, 86] and the visual cortex [87, 88]. Moreover, retinal projections to the SC have been identified in human embryos and fetuses [85]. In macaque monkeys, all early visual areas of the cortex, including V1, V2, V3, V4, and the middle temporal visual area, project to the SCs [87]. In addition, SC subpopulations receive direct inputs from the ipsilateral V1 and surrounding regions in other mammals [46, 89–94]. A number of features of the primate SC suggest that it processes visual information differently from the mouse SC. In primates, SC neurons respond to visual stimuli within their receptive field (RF) regardless of the specific features of the stimulus. This type of neuron is often called an event detector. Event detector cells are the most numerous in the superficial primate SC and are not selective for specific directional movement, orientation, or shape [95–98]. These neurons are suited to encoding the location of visually salient novel objects. Approximately 10% of RGCs project to the SC in macaques [86], whereas ~90% of RGCs project to the SC in mice [99]. It is possible to explain a conserved SC network involving visual motion processing from rodents to primates. In macaques, the frontal eye field (FEF) [100], a visuomotor area in the prefrontal cortex, and the supplementary eye field [101], a cortical area within the medial frontal area, send strong projections to the ipsilateral SCi. The SCi regulates saccadic eye movement, whereas the SCd receives dense projections from the primary and supplementary motor cortices [102]. The SCi and the SCd function as a midbrain sensorimotor structure and receive a moderate projection from the dorsal and ventral premotor cortex, the anterior and lateral intraparietal cortex, the inferior parietal cortex (inferior parietal lobule area 7b), and the ventrolateral prefrontal areas [103–105], forming a network involved in the control of purposeful hand actions. In rhesus monkeys, cells in rostrolateral regions of the SNr project largely to lateral portions of the SC [106]. Considering the large number of inputs to the colliculus, there are few studies demonstrating the precise relationship between these inputs and neuronal populations.

#### SC Inputs in Non-primate Mammals

Studies investigating SC circuits in non-primate mammals mainly involve rodents, but there are some which investigated tree shrews [107] and cats [108], and revealed cortical efferents to the SC. Tree shrews, with a direct phylogenetic relationship with primates, have visual cortical projections to the SC [107]. In cats, the SC also receives cortico-tectal projections from the visual, auditory, somatosensory, motor, and limbic cortices; however, the majority originate in the visual cortex [108].

In mice, SCi and SCd neurons receive projections from the auditory cortex [21, 109], the cingulate cortex, the primary and secondary somatosensory cortices, the parietal association cortex, and the agranular insular cortex [22]. Although the architecture of the mouse SC is similar to that of primates, the visual response properties of mouse and primate SC neurons are different. Mouse SC neurons act more like ‘‘feature detectors’’ in that a specific subset of SC neurons responds best when a specific type of stimulus is presented within its RF. These neurons might be useful for detecting the visual features of a potential threat and immediately respond by initiating a defensive behavior without further analysis of the visual scene [19, 28, 44, 110–113].

In addition, the SC receives inputs from several subcortical regions. In rats, SCs neurons receive direct inhibitory inputs from the ipsilateral pretectal nuclear complex (the visual and visuomotor control structure) [27]. Also in rats, 5-HT-positive projections to the SC arise exclusively from the nuclei raphe and the contralateral periaqueductal grey [114]. In addition, GABAergic and glutamatergic neurons in the SCi and SCd both receive GABAergic inputs arising from the SNr in rats and mice [26, 115]. In mice, the SCs receives inputs from nearly every RGC [99]. Further investigation has demonstrated that the precision of retinotectal signaling is highly dependent on intrinsic GABAergic circuits [24, 54, 111, 116] and approximately one third of the postsynaptic targets of retinotectal terminals are GABAergic [117]. Neurons in the SCi and SCd also receive projections from the cerebellar nuclei, the pedunculo-tegmental nucleus, the ventral posteriomedial thalamic nucleus [22], and the brachium and external cortex of the inferior colliculus [118], as well as dense fibers from cholinergic neurons in the parabrachial nucleus (PB) [25], dopaminergic afferents from the ZI [70] and tyrosine hydroxylase-positive afferents from the locus coeruleus [69]. That the SC receives significant inputs from visual, auditory, and somatosensory neurons in primates, cats, tree shrews, and rodents, confirms that the SC is a conserved sensorimotor structure that integrates visual and other sensory information to drive various behaviors. Future studies using modern molecular and genetic methods have the potential to exemplify the evolution and conservation of the SC neuronal cell types and circuits that give rise to specific behaviors.

#### Optic Tectum Inputs in Lamprey and Zebrafish

Compared to the SC, which lies under the cerebral cortex in mammals, the OT (homolog of the SC) in non-mammals tends to lack cerebral cortex coverage [35]. The visual cortex is less prominent in non-mammals, and is even absent from lower vertebrates such as fish. For instance, RGC axons terminate in the OT in zebrafish [119, 120], revealing an evolutionally conserved retinal pathway that conveys visual signals involving the SC/OT. In addition to RGCs, the OT receives projections from several subcortical regions, including the substantia nigra pars compacta (SNc) in lampreys [121]. In zebrafish [122], the SNc sends abundant dopaminergic innervation to the deep layer of the OT with sparse projections to the superficial layer. In larval zebrafish, the deep OT receives inhibitory projections from the ventral thalamus, which detects a decrease in the luminance signal and delivers dim-specific information to the OT, and hence drives directional startle [123]. However, the efferent axons from ventral thalamic structures present in zebrafish and amphibians have not been found in mammals [124]. The absence of a homologous brain projection in mammals may explain why the looming-detection role of the ventral thalamus in zebrafish has been reported less often in rodents. In larval zebrafish, inhibitory projections from the rostral hypothalamus specifically target the deep retinorecipient laminae of the tectal neuropil in addition to non-retinorecipient laminae [125]. However, functional analyses based on current data are unable to discriminate whether the rostral hypothalamic projection is a direct monosynaptic connection or dependent on the activity of intermediate structures. Further, while issues regarding the identity, morphology, connectivity, and function of the hypothalamus–OT pathway remain, it has been proposed that the rostral hypothalamic projection feeds directly into the tectal circuit. In addition, the nucleus isthmi, which is thought to correspond to the parabigeminal nucleus (PBGN) in mammals, forms reciprocal connections with the OT, modulating tectum-dependent goal-directed behavior [126].

As noted above, an evolutionally conserved pathway between the retina and the SC has been demonstrated by the connectivity patterns in humans, monkeys, rodents, and zebrafish. Overall, more than a dozen input pathways to the SC/OT have been identified. Interestingly, the selective activation of many of these pathways leads to divergent responses, such as visual processing, sleep, and defensive behavior.

### Overview of Outputs from SC Neurons (Figs. 1 and 2)

#### SC Outputs in Humans and Non-human Primates

The development of optogenetic approaches in non-human primates has given rise to speculation that these methods may one day be feasible for use in human studies [127]. However, in part because of the ethical challenges associated with direct manipulation and neuronal recording in the human brain, neuroimaging in human neuroscience has lagged behind that of animal work. Thus, fMRI will likely remain the principal neuroimaging method in human studies for the foreseeable future [128]. The SC projects to the pulvinar, a structure homologous to the mouse lateral posterior thalamus, in both rhesus monkeys [129] and humans [129, 130]. In rhesus monkeys, the SC sends projections to the central mesencephalic reticular formation [30], the ipsilateral PBGN [131], and the SNr, in addition to brainstem regions including the dorsal lateral pontine gray, the nucleus reticularis tegmenti pontis, and ipsilateral deep tectal layers of the cuneiform nucleus (the external nucleus of the inferior colliculus) [132]. In prosimian primates, SC projections also terminate in the pulvinar complex [133]. Interestingly, the SC not only receives dense afferents axons from the FEF [100], but also sends feedback to the FEF *via* the mediodorsal thalamus in *Macaca mulatta*, conveying a corollary discharge related to saccades [134]. Moreover, a direct projection from the SCs to the lateral geniculate nucleus has been identified in both owl monkeys [135] and marmosets [136]. In owl monkeys, there are projections from the SC to the K2 and K3 layers of the lateral geniculate nucleus [135]. In addition, in *Macaca*, SC neurons project to the ipsilateral PBGN, and the PBGN sends dense cholinergic projections to both the ipsilateral and contralateral SC as feedback pathways [131, 137], similar to the reciprocal connection between the SC and the PBGN in cats [138], and similar to the OT and the nucleus isthmi (thought to correspond to the PBGN in mammals) in zebrafish [139, 140]. These basic similarities in this parabigemino-tectal circuit between a wide variety of vertebrates raises the possibility that, through the course of evolution, it has come to mediate a similar visual-processing function across all species.

#### SC Outputs in Non-primate Mammals

There has been much investigation of SC outputs in non-primate mammals, especially in rodent models, that may facilitate our understanding of the neural architecture of the SC in humans. A careful review of the SC output literature reveals an interesting kernel: terminals from ascending projections are more extensive and intensive than are the descending projections from the SC to the pons. In rats, the SCi provides projections to the thalamic reticular nucleus [141], the brainstem reticular formation including the paramedian region of the pontine reticular formation, the tegmental reticular nucleus, the gigantocellular and medullary reticular nuclei, and the parvocellular and peri-abducens regions, as well as a number of pontine and medullary sites including the pedunculopontine nucleus, the laterodorsal tegmental nucleus, and the facial nucleus [142]. The SCd sends a strong, region-specific projection to the subthalamic nucleus [143, 144]. Moreover, the SC projects to the thalamic reuniens nucleus [145], the SNc [91], the brainstem pre-Bötzinger complex [146], and the cuneiform nucleus [147], and projects excitatory innervation into the ZI [148].

In mice, GABAergic [15] or PV-expressing excitatory neurons in the SCs [45, 46] innervate the pretectum, lateral geniculate nucleus, and PBGN. A projection from the PBGN to the SC has also been found in monkeys [131]. In addition, vGat-expressing neurons in the SCs form monosynaptic functional connections with VTA dopaminergic neurons [24]. Moreover, SCi and SCd glutamatergic neurons project to the VTA [28] and the lateral posterior thalamus [31], which are both crucial for visually-evoked innate fear behaviors. Further, excitatory subcortical neuronal circuits have been uncovered from the SCi and SCd to GABAergic ZI neurons [29] and to dorsal periaqueductal grey neurons [32].

It has been shown that SCi neurons and oxytocin neurons in the paraventricular nucleus of the hypothalamus are reciprocally connected [149]. In mice, SC projections to the different cell populations of the laterodorsal tegmental nucleus, including glutamatergic, cholinergic, PV^+^-GABAergic, and SST^+^-GABAergic neurons [150], and to the mediodorsal thalamus, are indispensable for preventing the retrieval of fear memory [151]. Compared to the SC–mediodorsal thalamus–FEF pathway involved in saccadic eye movements in monkeys [134], the above findings suggest that it is not yet known whether specific-cell-type SC–mediodorsal thalamus connections execute different behavioral outputs. In addition, there are distinct inputs in mice from the SC to the cuneiform nucleus, the anterior pretectal region, and the inferior olivary complex [46]. Furthermore, a very recent study systematically delineated the distinctive brain-wide input/output organization of the SC and identified cortex–SC–thalamus and SC–brainstem subnetworks, which taken together consist of sensory-motor and polymodal association thalamus and provide comprehensive SC afferents and efferents [21]. Future functional studies of SC input/output pathways should contribute to identifying associated physiologically-relevant behaviors.

#### OT Outputs in Zebrafish

In zebrafish, the single-cell atlas of tectofugal neurons has been used to identify axonal projections from the tectum to promotor areas. Among the OT recipient zones are areas situated in the pretectum, thalamus, contralateral tectum, ipsilateral tegmentum, medulla oblongata, and ipsilateral and contralateral reticular formation [152], which together transform visual inputs into directed swimming behavior. However, the cell-type specificity and synaptic transmission of these connections need further investigation.

In summary, connectivity between the SC/OT and thalamus, which exists in primates, rodents, and zebrafish, and known to be involved in the vision-related information processing in many species, is highly conserved across species. However, neuronal subpopulations in SC subregions, and their cell-specific projections and physiological functions need further characterization.

### Overview of intrinsic SC circuits

Intrinsic SC circuits include the intercollicular commissure and the tecto-tectal commissure. The SC on each side are interconnected *via* the intercollicular commissure across species, including humans [153], non-human primates [132], cats [154], and rodents [155, 156]. It has been proposed that this circuit mediates visual transformation and gaze orienting, while the inhibitory commissural pathway has been confirmed in rats, cats, and pigeons [157–159] and is thought to be responsible for reciprocal inhibition.

Wurtz and Mohler initially established in primates a visual enhancement effect in the ipsilateral SC, supporting evidence of an intrinsic connection between superficial and intermediate layers [160] or between cortical inputs. It was shown subsequently, in humans, that the SC processes intrinsic connections within the SCs and connections from SCs to SCd [161]. Moreover, cats have a tecto-tectal excitatory commissural pathway in the SC [61]. The putative roles of these intrinsic connections include visual receptive field organization as well as visuomotor and multisensory integration. In mice, the SCs receives GABAergic input from the SCi and SCd and provides powerful excitatory input to premotor neurons in the SCi and SCd [56, 73]. In contrast to feedforward pathways that translate sensory information into motor commands, a feedback pathway from excitatory SCd motor neurons to SCs sensory neurons has been revealed in rat SC slices [59], together with a glutamatergic excitatory pathway from the SCs to the SCi [162]. These internal circuits within the SC are important for understanding functions involving the SC.

### Projections without Identified Directionality in Humans and Non-human Primates

The SC detects, distributes, and integrates ascending and descending information to and from various brain areas. Connections between the motion-sensitive cortex (middle temporal visual area) and the SC have been identified in humans [163]. In addition, in patients with the dissociative subtype of post-traumatic stress disorder, functional connectivity has been shown to be elevated between the right SC and the right temporo-parietal junction (TPJ), and between the left SC and the right dorsolateral prefrontal cortex [164]. These findings contribute to understanding of the SC as a threat-detection hub of the innate alarm system. Furthermore, a functional connection between the superior temporal sulcus, a presumptive homolog of the TPJ, and the SC, has been found in monkeys [165]. Moreover, a human study investigating bodily illusions showed increased functional connectivity between the SC and the right TPJ, bilateral ventral premotor cortex, and bilateral postcentral gyrus [166]. However, such functional neuroimaging studies are occasionally criticized for being purely correlative, and therefore uninformative regarding underlying mechanisms. Task-based fMRI has been responsible for much of the recent increase in human neuroscience research. Dissecting these task-based associations between brain areas may provide evidence to facilitate our understanding of the pathogenesis underlying associated diseases and the search for possible intervention targets.

### A Comparative Perspective of SC Conservation Across Species

As described above, there are retinal projections to the SC in humans, monkeys, mice, and zebrafish and connections from the visual cortex to the SC in humans, monkeys, and mice; together, these findings reveal an evolutionarily conserved pathway used to convey visual signals. A reciprocal connection between the ZI and the SC has been identified in macaques, squirrels, and mice [70, 167], indicating a conserved pathway between the ZI and the SC. Connections between the motor cortex and the SC in monkeys and mice, as well as between the SNr and the SC in monkeys and mice have also been identified, and are posited to mediate goal-directed behaviors. SC neurons project to the PBGN, a connection that is similar to the reciprocal connection between the OT and the nucleus isthmi (thought to correspond to the PBGN in mammals) in zebrafish. Direct projections from the SC to the lateral geniculate nucleus have been identified in monkeys and mice. Connectivity between the SC and the pulvinar in rhesus macaques and humans (a structural homologue of the lateral posterior thalamus in mice) has also been demonstrated, together with the precise location and function of a pathway found in humans from the SC to the amygdala *via* the pulvinar, revealing that it encodes aversive auditory and visual (but not painful) stimuli, supporting unconscious affective responses [130]. Tied together with the SC projection to the lateral posterior thalamus in mice, which modulates threat-related behavior, it appears that a conserved circuit from the SC-pulvinar/lateral posterior thalamus exists across species, although comparative investigations of specific cell types and target laminae in rodents and primates are required.

Basso and Krauzlis [35, 37] highlighted possible reasons why the SC is considered to be highly conserved across species. First, the SC consists of alternating fibrous and cellular layers, topographically mapped to allow the integration of multisensory information and behavioral initiation. Second, different species share some of the same neuronal architecture. For example, ancient circuits that have a common structure and function are conserved, although evolution has shaped neural circuits to give rise to species-specific behavior.

In summary, the SC pathways conserved through evolution generally occur across species. Common neural circuits are considered responsible for similar behavioral outputs. Neurons originating from different progenitor pools form complex but precise neural circuits, and diversification of neuronal types in neural circuits govern functional variation. However, cell-type-specific neural circuits are difficult to define in human and non-human primates owing to limited technology, leaving a long-lasting question about the extent to which neural circuits and functions are common among different species. The challenge moving forward is to understand the function of the interactions between the SC and the forebrain and how these interactions evolved from non-mammalian vertebrates to mammals, including primates.

## SC circuits underlying behavior

Studies that applied Ca^2+^ imaging, chemogenetic and optogenetic studies [29], electrophysiological recording, functional ultrasound imaging [168], and high-throughput and circuit-specific single-cell transcriptomic analyses [19] have led to the conclusion that certain behavioral responses are initiated or modulated by the SC. Here, we focus on SC circuits in eye movement, cognitive behavior, and innate behavior, including visually-evoked innate fear, prey-capture, drinking behavior, and sleep.

### SC Circuits and Saccadic Eye Movements (Fig. 3A)

**Fig. 3:**
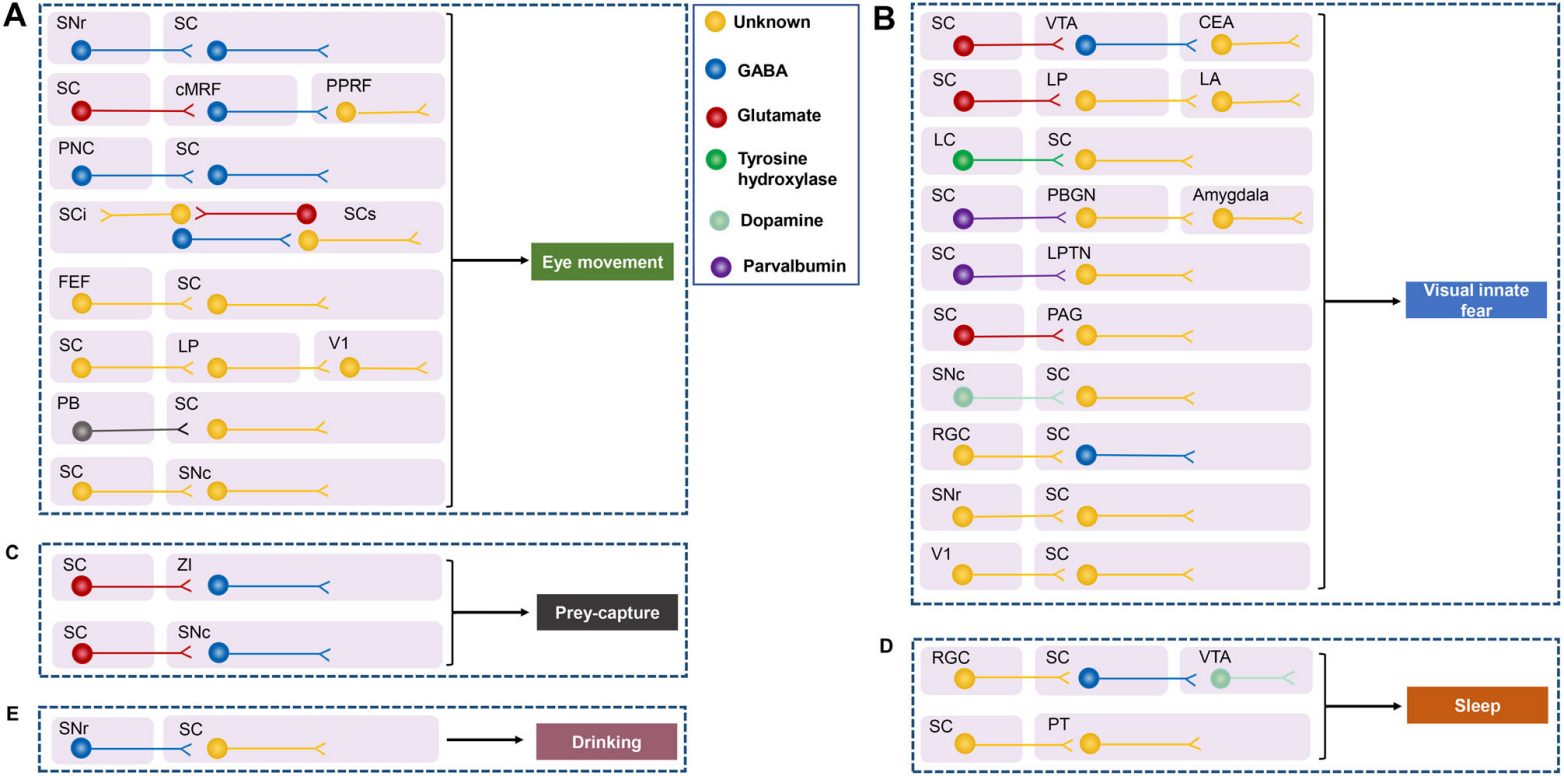
Contributions of specific superior colliculus circuits to eye movements and innate behaviors. **A** Schematic of the inputs onto GABAergic and glutamatergic SC neurons proposed to contribute to eye movements and salient vision. **B** Schematic of the sources of glutamatergic inputs synapsing on zona incerta (ZI) GABAergic neurons shown to participate in prey-capture behavior [hunting test with the introduction of a cockroach (prey) to a mouse (predator) in a confined arena]. **C** Schematic of the inputs to GABAergic SC neurons proposed to contribute to wakefulness (an acute pulse that increases wakefulness in nocturnal animals). **D** Schematic of inhibitory inputs from the SNr to SC neurons proposed to contribute to drinking behavior.** E** Schematic of the glutamatergic, parvalbumin-positive, or tyrosine hydroxylase-positive inputs to the SC that have been proposed to contribute to visual fear behavior [an animal is exposed to an expanding dark disc (looming) stimulus to the upper visual field to mimic an approaching aerial predator]. VTA, ventral tegmental area; CEA, central medial amygdala; LP, lateral posterior thalamus; LA, lateral amygdala; LC, locus coeruleus; PBGN, parabigeminal nucleus; PAG, periaqueductal grey; SNc, substantia nigra pars compacta; RGC, retinal ganglion cells; SNr, substantia nigra pars reticulata; V1, primary visual cortex; PT, pretectum; cMRF, central mesencephalic reticular formation; PPRF, paramedian pontine reticular formation; PNC, pretectal nuclear complex; SCi, intermediate gray layer of the SC; SCs, superficial layer of the SC; FEF, frontal eye field; PB, parabrachial region.

Saccadic eye movements enable fast and precise scanning to visual targets to resolve high-acuity images. In 1971, Wurtz and Goldberg demonstrated that neurons in the SC always discharge prior to saccadic eye movements and could thus be logically related either to the initiation of eye movement or to a discharge corollary to eye movement in awake monkeys [169], and to saccadic eye movements that rapidly shift the gaze of the animal toward an interesting object so that the animal can visualize it in greater spatial detail with its fovea [170, 171]. In support, it was well demonstrated that the SC is essential for the generation and control of saccadic eye movements in humans [2], monkeys [171–173], cats [174, 175], mice [73, 115], rats, and hamsters [176]. Although the SC is used to control eye movements in mice, its purpose is unclear, as they do not have a fovea. In macaques, the SC provides input to central mesencephalic reticular formation neurons, within which circuits code for saccade signals [177], while optogenetic stimulation of the FEF–SC pathway evokes saccadic eye movements in monkeys [100]. The SNr provides monosynaptic inhibition to GABAergic neurons in the SCi, which modulates premotor neuron properties and saccade initiation [42], while the SCi receives dense cholinergic innervation from the parabrachial region of the brainstem [25], which may influence the execution of orienting movement behavior in mice. Further, a GABAergic feedback circuit from the SCi to the SCs may suppress eye movement [73]. Moreover, optogenetic modulation of the mouse LP has shown that the retina–SC–LP–V1 pathway forms a differential circuit to the canonical retino-geniculate pathway to achieve context-dependent sharpening of visual representations [178]. In rats, glutamatergic SCs inputs to the SCi and the SC projects directly to the SNc, and these are involved in saccade control [162] and in the detection of salient visual events [91], respectively. GABAergic projections from the pretectal nuclear complex target the SCs, a central structure for visual information processing necessary for the generation of saccadic eye movements [27]. Together, these findings indicate that the SC is essential for the execution and modulation of eye movement.

### Higher Cognitive Function: Target Attention and Decision-making

#### The SC and Attention

In 1890, the psychophysiologist James described attention as the ability to selectively filter perceptual information combined with reorientation towards the object of interest, while ignoring other information and gradually bringing the object from unconsciousness into awareness [179]. The large-scale network of brain regions responsible for attention comprises the neocortex, including the visual [180, 181] and parietal cortices [182]. Beyond these cortical areas, Goldberg and Wurtz put forward the hypothesis that the SC is also involved in visual spatial attention. The SCs response to visual stimulation is enhanced when monkeys made saccades to targets in their receptive fields, compared to when they their eyes remain fixated in the presence of the targets [95]. The central role of the SC in visual spatial attention has been defined in previous decades [35, 37, 183–186]. For example, recordings of single-cell activity in the SC of monkeys trained to perform a series of visuo-oculomotor tasks suggest that tonic activity in the SC is related to peripheral attention [187]. Moreover, low-intensity electrical stimulation of the SC, which does not evoke saccadic eye movements, enhances motion-direction discrimination [188] and detection [189, 190]. More importantly, Lovejoy and Krauzlis [185] demonstrated perhaps the most convincing causal link between the primate SC and attention. They teased apart selective attention deficits from local visual deficits and showed that the deficits did not depend on saccadic eye movements. It is possible that, with the exception of the direct role in influencing attention, SC activity also contributes to the activation of other brain regions, and hence orchestrates neuronal network dynamics to control attention and behavioral output. Perturbation of SC activity significantly alters attention-related visual processing in the basal ganglia, specifically in the head of the caudate nucleus in the striatum, which infers that interaction between the SC and the caudate nucleus confers normal visual attention [43]. In addition, a recent study in mice identified an inferior colliculus–SC circuit that controls auditory cue-directed visual spatial attention [186]. In summary, at least in non-human primates and rodents, it is an established fact that the SC plays a causal role in attention.

#### The SC and Decision-Making

Understanding how the brain makes decisions is a major area of focus in both animal and human cognitive neuroscience. The ability to make decisions allows complex nervous systems to flexibly guide appropriate responses that are adaptive to the environment, and hence decision-making is thought of as one of a few fundamental forms of cognition. Glimcher and Sparks were the first to report that the SC participates in the saccade selection process [191]. Basso and Wurtz also reported that the SC may play a role in decision-making after recording from monkeys during a decision-making task during which they found that delay-period signals recorded in the SC were correlated with the certainty of an impending saccade rather than being obligatorily linked to eye movement [192, 193]. It is also known that unilateral inactivation of the SC in non-human primates following microinjections of lidocaine or muscimol alters target selection or perceptual decision-making in a two-choice perceptual decision-making task. This adds to the accumulating evidence suggesting a causal role for the SC in decision-making, in addition to the classical notion of being involved in simpler functions related to the control of saccadic eye movements [36, 194–197]. In one study, electrical manipulation of non-human primate SC neuronal activity changed decision outcomes predictably, demonstrating a causal role of the SC in decision criteria [198]. Similarly, results following transient unilateral inhibition of the SC achieved *via* targeted optogenetic activation of GABAergic inhibitory neurons that induce specific spatial deficits, strongly suggest that the SC plays a causal role in a visual perceptual decision-making task in male and female mice [39]. Very recently, long-range projections from GABAergic SC neurons, but not from local projections, were found to mediate decision-making in mice [18]. These studies suggest that the SC regulates decision-making behavior in both rodents and non-human primates. These new results indicate that the mouse and primate SC is necessary for normal performance during voluntary visual choice behavior and highlight the necessity to explore the circuits ascending from the SC to the forebrain, circuits composed of specific cell types of which relatively little is known.

### SC Circuits and Innate Behaviors (Fig. 3)

Given its access to primary sensory signals including visual and somatosensory cues, the SC appears to rapidly process and transmit information regarding stimulus characteristics to related brain areas, which are then used to modulate behavior. To answer the core question of how neural circuitry affects behavior, it is of great important to systematically map functionality onto specific anatomical connections of the SC. Over the past decade, investigation of SC function has expanded from saccadic eye movements to behavior motivated by survival instincts. Rapidly-evolving technologies, such as optogenetics, chemogenetics, two-photon imaging, and fiber photometry, in conjunction with behavioral observations and electrophysiological recordings, have enabled researchers to reveal casual relations between specific neural circuit and innate behavior. The use of robust experimental paradigms, such as looming, which evokes an innate fear response, or predatory hunting, have revealed that the SC plays a crucial role not only in saccadic eye movement but also in behaviors with a tight stimulus-response. These are evolutionally conserved behaviors guided by seeking profit whilst avoiding injury, such as visually-evoked innate behaviors and prey-capture behavior. These types of studies provide evidence which aids our understanding of the evolutionarily conserved characteristics of the SC, serving as a sensorimotor hub, allowing functional findings from one species to inform knowledge of others.

#### The SC Mediates Visually-Evoked Defensive Behaviors Across Species (Fig. 3B)

When we are faced with a potential life threat, the faster we can perceptually discriminate, the sooner we can initiate a potentially life-saving defensive response. This raises a question of how sensory information first reaches brain regions that elicit crucial responses to life-threatening stimuli. To assess visually-evoked defensive behaviors in the laboratory, looming visual stimuli pose an evolutionary threat to animals, as they signify the presence of flying predators across species [122, 199–202]. A pioneering study in rats revealed that lesions of the SCd attenuate fear-potentiated startle, a reflexive action to an acoustic stimulus previously paired with a shock, which is then presented in the presence of light to evoke a startle [203]. In mice, SCs neurons respond to many different kinds of visual display but only in the sharp receptive field, while SCd neurons are selective for looming stimuli, exhibiting characteristics of invariance to stimulus position and habituation to familiar stimuli [14], suggesting that SC neurons are capable of both visual signal detection and discrimination.

How is the looming-evoked defensive behavioral expression pattern guided by specific SC neural circuitry? Looming stimuli of different speeds elicit variable responses, with fast presentations driving short-latency escape or freezing behavior and slower presentations producing longer-latency escapes that are more kinematically variable [204]. Moreover, it has been shown that subcortical pathways originating from the SC have genetically identifiable cell types that process visual life-threatening information, and causally and independently evoke distinct defensive responses. A growing body of optogenetic and neuroanatomical tracing evidence in mice has partially clarified the link between specific neural circuits and behavioral expression during the looming paradigm. For instance, activation of the glutamatergic SC projection to the lateral amygdala *via* LP circuitry is important for freezing behavior following looming stimuli [31]. Moreover, two distinct groups of PV^+^ neurons in the SC that deliver threat-relevant visual signals to PBGN and LP neurons, induce flight and freezing behavior, respectively [44, 45]. Furthermore, monosynaptic connections between glutamatergic dorsal medial SC and dorsal periaqueductal grey neurons function as a synaptic threshold for decisions to escape overhead expanding spots [32]. A glutamatergic SC projection to the amygdala *via* GABA^+^ VTA neurons to the central nucleus mediates visually-evoked flight behavior [28]. These results indicate that SC neurons determine the defensive behavioral expression pattern before visual signals have been transferred to downstream areas.

A recent fundamental study using high-throughput single-cell transcriptomics combined with optogenetics has provided evidence for the involvement of distinct layer-specific glutamatergic SC subpopulations (Cbln2- or Pitx2-positive) and separate circuits during vision-related defensive behavior or hunting behavior [19], posing the question of how SC neurons integrate information and whether they directly determine behavioral expression patterns. In addition to extensive projections arising from the SC, the SC receives axonal projections that mediate a complex set of looming-evoked defensive behaviors. For instance, the TH^+^ locus coeruleus neurons projecting to the SC mediate stress-induced accelerated looming-evoked defensive behavior [69], while V1 neurons projecting to the SCs modulate looming-evoked responses in awake mice [112]. Altogether, these studies from rodent research show that different types of stimulus and/or environmental context govern different behavioral outcomes, and that the SC plays a central role in parallel subcortical pathways using information arising from multiple sources and, in addition, that the SC plays a central role in processing optimal dimorphic defensive behaviors from the earliest stages of visual processing. Uncovering cell-type-specific modulation *via* the SC may provide evidence to further our understanding of the balance between excitation and inhibition for “gating” the relaying of threat signals.

There is also accumulating evidence that the SC/OT is activated in response to unexpected aversive events and looming stimuli to initiate defensive behaviors in other species, including humans [200, 205], monkeys [201], cats [202], pigeons [206], amphibians [207, 208], and zebrafish [122, 209, 210].

The characteristics of SC/OT neurons that respond to visual signals and the associated functional properties are not totally consistent between zebrafish and mice, which can be partly attributed to the structural specificity of laminae [35, 211] and a different source of RGC inputs [212, 213]. For example, there are edge-sensitive, dim-sensitive, looming-specific neuronal populations in the zebrafish OT [123]. A distinct neuronal population map of the OT has been built using a linear combination of diverse, functionally specialized, lamina-specific, and topographically-ordered RGC inputs, which appear to contribute to the function of visual-signal detection and discrimination in the OT [212]. Another study delineated a topographical map of a subset of superficial interneurons in the zebrafish OT, among which most expressed a GABAergic marker and were strongly responsive to changes in whole-field luminance [214]. RGC projections to the OT in zebrafish are required for visually-evoked escape [122, 209], while thalamic projections to the OT relay luminance information and facilitate looming-induced escape responses [123]. Moreover, dopaminergic SNc projections to the OT modulate distinct looming-induced motor responses in lampreys [215]. Furthermore, a recent fundamental study provides compelling evidence for the involvement of distinct SC neuronal populations and includes a separate circuit from the lateral posterior thalamic nucleus and ZI projecting to SC neurons that influence different behaviors [19] In summary, these findings indicate that diverse cell populations and neural circuits involving the SC (or OT) determine different defensive strategies.

#### SC Circuits and Prey-Capture Behavior (Fig. 3C)

Prey-capture behavior is an evolutionarily conserved appetitive behavior predominantly mediated by prey-detection signals. Rodents are both predators and prey; they hunt cockroaches [29, 47, 216] and crickets [217], and both audition and vision are used for accurate cricket approach and capture. In rats, the SC plays vital roles in anti-predator defense and hunting behavior. Loss of vibrissal input to the SCi following whisker removal has revealed a critical role of somatosensory inputs to the SC in predatory behavior [47]. In mice, wide- and narrow-field vertical neurons in the SC are differentially involved in distinct aspects of prey capture [217]. Wide-field neurons are required for rapid prey detection and distant approach initiation [218], while narrow-field neurons are required for accurate orienting during pursuit in addition to approach initiation and approach continuity [217]. A robust pathway from the SC to GABAergic ZI neurons provokes prey-capture behavior [29, 48, 216], suggesting that the SC and ZI serve as a critical integrative node that orchestrates sensorimotor or motivated information cooperatively to give rise to multiple behaviors. Similarly in mice, activation of the excitatory brain circuit from the SC to the SNc promotes appetitive locomotion during predatory hunting [219], while the photoactivated SNr projection to the SC leads to an increase in defensive behavior to the approach of a moving robo-beetle [220].

In larval zebrafish, the size of moving objects in the visual field determines either hunting or escape behavior (small object, potential prey; large object, potential predator) [210, 221]. Superficial OT interneurons serve as motion detectors for sensing, localizing, and determining the size of moving objects within the visual field [210]. It also has been demonstrated that inhibitory [222] and glutamatergic neurons [221] within the zebrafish OT provide size tuning that distinguishes prey *versus* predator stimuli. Moreover, either photo-ablation of OT cells or silencing of synaptic transmission in the OT eliminates the size tuning associated with the deeper layers and impairs capture of prey [223]. In both rodents and larval zebrafish, the SC/OT facilitates prey-detection signals (visual, olfactory, auditory, and somatosensory), and in turn, integrates information to initiate responses towards prey through descending pathways [29, 47]. These findings add perspective required for understanding the function of SC/OT neurons. However, there are still some questions to be answered. For example, how much and what aspects of this behavior depend on the SC or the cortex? Where do the locomotion-speed signals of the SC/OT originate? How does the SC/OT distinguish behavioral actions during predatory hunting? Is there a shared SC/OT pathway that modulates predatory hunting across species?

#### SC Circuits and Sleep (Fig. 3D)

Sleep is a readily reversible state of reduced responsiveness, reduced motor activity, and reduced metabolism [224], which contributes to restoring energy in vertebrates and invertebrates, and generally involves stages termed rapid eye movement (REM) sleep and non-rapid eye movement (NREM) sleep. It has been shown that ablating the SC–pretectum attenuates acute sleep-waking responses to changes in lighting conditions, including the triggering of REM sleep after light-off stimulation and redistribution of NREM to the light periods of short light-dark cycles [218]. In addition, c-Fos expression and non-cell-specific lesion data suggest that the SC plays an important role in acute light induction of sleep [218, 225]. The firing rate of SC neurons is higher during REM sleep than in periods of awake immobility and slow-wave sleep [49]. Several studies have determined that specific SC cell types are involved in sleep induced by acute light [24, 218, 225]. A combination of optogenetics, electrophysiology, and selective lesion experiments have uncovered a fundamental role of a retinal-SC–GABAergic-VTA dopaminergic circuit in acute dark induction of wakefulness in mice [24]. Also in mice, direct photic regulation of sleep is predominantly mediated by the melanopsin-based photoreception of photosensitive retinal ganglion cells, involving the direct activation of specific sleep-promoting centers of the brain, including the SC [225]. As noted before, the SC sends direct projections to the thalamic reticular nucleus [141], which acts as a key player in sleep control [226], indicating that the SC → thalamic reticular nucleus pathway may also play a fundamental role in both sleep and wakefulness. These findings suggest that SC circuits likely play a more important role in sleep and arousal than currently recognized.

#### SC Circuits and Drinking (Fig. 3E)

Consummatory behaviors, such as drinking, are critical for individual homeostasis and survival [227]. In rodents and other mammals, drinking is achieved by stereotyped and repetitive licking movements at a relatively constant frequency. Bilateral injection of the GABA agonist muscimol into the SC produces stereotyped gnawing and biting during drinking, suggesting that the SC is important for orofacial behaviors during drinking [228]. Furthermore, selective optogenetic activation of GABAergic nigrotectal afferents in the SCd [229] or inhibition of the SC in mice both result in the inhibition of licking behavior [39]. As such, careful investigation of distinct cell subpopulations within the SNr and SC is warranted to resolve the contributions of different neuronal populations, which may explain drinking and orienting movement behaviors stemming from the same neural circuit.

## The SC and Neurodegenerative/Neuropsychiatric Disorders

Considering the various physiological functions embedded in the SC, especially the perception of emotional signals, it is easy to speculate that dysfunction of the SC is involved in pathophysiological processes [230]. Disturbance of the SC is linked to sensory processing disorders, such as autism spectrum disorder [231] and fragile X syndrome [232], neurodegenerative psychiatric disorders, including Parkinson’s disease (PD) [233–235], dementia with Lewy bodies [236] and neuropsychiatric disorders, such as attention-deficit hyperactivity disorder [237], and epilepsy [238–242]. For example, in autistic children presenting abnormal defensive responses to looming stimuli, structural connections in the SC–pulvinar–amygdala circuit are weaker than expected [231]. Taken together with evidence that the SC–pulvinar–amygdala pathway encodes negative emotion in healthy volunteers [130] and that the SC–lateral posterior thalamus circuit in mice modulates defense behavior evoked by a threatening signal [19, 31, 44], it is reasonable to speculate that the conserved SC–pulvinar–amygdala pathway may underlie dysfunction of perception in autism, at least in part. In patients with PD, the SC is involved in both visual and motor dysfunction, including dysfunctional saccades [243, 244], deficient motion perception and performance [244, 245], impaired emotional face perception and rapid response [246], reflex blink hyperexcitability [247], and an impaired luminance contrast response [248], which are consistent with previous understanding of the SC as a conserved sensorimotor structure. Interestingly, the SC may also play a role in seizure control by exerting anticonvulsant effects *via* connections with the basal ganglia system, particularly the SNr [249–251], the inferior colliculus [252, 253], and the thalamus [254], suggesting that epileptiform activity might involve the SC, although further studies are required to determine any causality. In addition, the SC is a major locus of interest for potential therapeutic targets in treating hyper-responsivity and distractibility in attention-deficit hyperactivity disorder in humans [255]. In summary, dysfunctional interplay both within internal SC circuits and the many SC inputs/outputs provides new insights into the pathogenesis of neurological deficits affecting a significant number of brain disorders.

## Conclusions and Perspectives

In this review, we have described SC neural phenotypes and associated cell-specific connections and circuits, and their contributions to eye movement and behavior. More than 40 identified molecules currently serve as valuable tools for the examination of mechanisms and connections between specific cell types that form defined circuits. Classical methods involving focused lesioning, electrical stimulation, and pharmacological approaches have raised questions about how distinct subregions and neuronal populations combine to functionally interact with other brain regions. The advent of transgenic mouse models and new molecular and optogenetic tools to identify and selectively manipulate selected neuronal cell types provide new insights linking different SC neuronal cell types to circuits that drive complex behaviors. The SC contains diverse neuronal populations and numerous inputs and outputs. Here, we also highlight the evolutionary comparisons of SC connectivity and function across species, bridging gaps in our current understanding of SC functioning. Many SC neurons also release neuroactive peptides (Table 1), adding another level of complexity to the circuitry and likely further contributing to various behavioral states. Future studies aimed at identifying distinct cell types within SC subregions using genome-wide association and RNA-sequencing are expected to generate sophisticated neuronal-behavioral classifications [19].

Advanced molecular and genetic methods combined with sophisticated innate behavioral paradigms make it possible to address how neuronal subtypes and circuits modulate specific behaviors. The SC is architecturally similar in primates and rodents and appears to exhibit conserved cellular and laminar connections, such as the ubiquitous existence of connections between the retina and the SC/OT, or the SC–pulvinar/lateral posterior thalamus across species. Neural circuits involving predominant SC subpopulations and specific innate behaviors, such as visually-evoked innate fear, exhibit generalizable properties shared by different species. In rodents, somatosensory signal processing in the SC mediates innate behavioral responses. In primates, the SC appears sufficient to process visual information to direct gaze and attention, although it is poorly interconnected with other somatosensory regions. It is possible that the primate neocortex is more devoted to decision-making, computation, and other higher-order functions [35], and that regulation of innate behavior by the SC is more complex. In this regard, future studies are necessary to determine the messages encoded in the firing patterns of distinct SC subpopulations across different behavioral states. Changes in SC activity underlying spatial and temporal integration and the intricate, specific connectivity of the SC, which contributes to its functioning, remains to be explored. Moreover, these findings also provide evidence supporting locally-generated feature representations in the SC [75], and lay the foundations of a mechanistic and evolutionary understanding of their emergence. The SC, thought to relay and integrate visual, auditory or tactile information across various cortical and subcortical regions, extends projections to the thalamus and hypothalamus, and these drive reflexive behaviors. Studies of the hypothalamus and its subregions, such as the ZI, place the hypothalamus at a crossroads where interoceptive and exteroceptive signals are relayed to brain areas governing motivation and avoidance behavior. Understanding how the interplay between the SC and the hypothalamus further integrate internal-state information and external stimuli to guide appropriate behavioral responses aimed at restoring homeostasis is an important goal of SC investigation.

In summary, despite tremendous progress, there is still much to learn. Understanding how neural circuits act within the SC will provide valuable insights into how the brain generates and controls behavior. Molecular details of how lamination in the SC is achieved are lacking, as is an understanding of the mechanisms used to align somatosensory and auditory maps with the visual map in the SC. The cell-type-specific neural circuit-based behavioral heterogeneity is likely a reflection of SC neuronal activity, with diverse phenotypic characteristics linked with distinct circuit and structural connections. Also missing is information about how auditory and somatosensory information is processed and integrated in the SC. Finally, it is important to determine the dysfunctional interplay both within internal SC circuits and in the many SC inputs/outputs. This will indubitably provide new insights into the pathogenesis of neurological deficits affecting a significant number of brain disorders, especially those associated with disordered sensory and cognitive processing. In these regards, understanding the intricacies of neuronal activity in the SC and its circuitry across species holds tremendous promise to elucidate conserved aspects of human neuropsychiatric disorders.

## Data Availability

The data that support the findings of this study are available from the corresponding author upon reasonable request.

## References

[1] Cang J, Savier E, Barchini J, Liu X (2018). Visual function, organization, and development of the mouse superior colliculus. Annu Rev Vis Sci.

[2] Savjani RR, Katyal S, Halfen E, Kim JH, Ress D (2018). Polar-angle representation of saccadic eye movements in human superior colliculus. Neuroimage.

[3] Basso MA, May PJ (2017). Circuits for action and cognition: a view from the superior colliculus. Ann Rev Vis Sci.

[4] Wurtz RH, Albano JE (1980). Visual-motor function of the primate superior colliculus. Annu Rev Neurosci.

[5] Casagrande VA, Harting JK, Hall WC, Diamond IT, Martin GF (1972). Superior colliculus of the tree shrew: a structural and functional subdivision into superficial and deep layers. Science.

[6] Lund RD (1969). Synaptic patterns of the superficial layers of the superior colliculus of the rat. J Comp Neurol.

[7] Mize RR (1988). Immunocytochemical localization of gamma-aminobutyric acid (GABA) in the cat superior colliculus. J Comp Neurol.

[8] May PJ (2006). The mammalian superior colliculus: laminar structure and connections. Prog Brain Res.

[9] Cotter JR (1976). Visual and nonvisual units recorded from the optic tectum of Gallus domesticus. Brain Behav Evol.

[10] Swanson N, Swanson LW. Cajal's histology of the nervous system of man and vertebrates. Oxford University Press, 1995.

[11] Nauta WJ, Van Straaten JJ (1947). The primary optic centres of the rat; an experimental study by the bouton method. J Anat.

[12] Nauta WJ, Bucher VM (1954). Efferent connections of the striate cortex in the albino rat. J Comp Neurol.

[13] Verhaal J, Luksch H (2013). Mapping of the receptive fields in the optic tectum of chicken (Gallus gallus) using sparse noise. PLoS One.

[14] Lee KH, Tran A, Turan Z, Meister M. The sifting of visual information in the superior colliculus. Elife 2020, 9. 10.7554/eLife.50678 PMC7237212 32286224

[15] Whyland KL, Slusarczyk AS, Bickford ME (2020). GABAergic cell types in the superficial layers of the mouse superior colliculus. J Comp Neurol.

[16] Sooksawate T, Isa K, Behan M, Yanagawa Y, Isa T (2011). Organization of GABAergic inhibition in the motor output layer of the superior colliculus. Eur J Neurosci.

[17] Mize RR (1992). The organization of GABAergic neurons in the mammalian superior colliculus. Prog Brain Res.

[18] Essig J, Hunt JB, Felsen G (2021). Inhibitory neurons in the superior colliculus mediate selection of spatially-directed movements. Commun Biol.

[19] Xie Z, Wang M, Liu Z, Shang C, Zhang C, Sun L*, et al.* Transcriptomic encoding of sensorimotor transformation in the midbrain. Elife 2021, 10. 10.7554/eLife.69825 PMC8341986 34318750

[20] Reinhard K, Li C, Do Q, Burke EG, Heynderickx S, Farrow K. A projection specific logic to sampling visual inputs in mouse superior colliculus. Elife 2019, 8. 10.7554/eLife.50697 PMC6872211 31750831

[21] Benavidez NL, Bienkowski MS, Zhu M, Garcia LH, Fayzullina M, Gao L (2021). Organization of the inputs and outputs of the mouse superior colliculus. Nat Commun.

[22] Doykos TK, Gilmer JI, Person AL, Felsen G (2020). Monosynaptic inputs to specific cell types of the intermediate and deep layers of the superior colliculus. J Comp Neurol.

[23] Savier E, Eglen SJ, Bathélémy A, Perraut M, Pfrieger FW, Lemke G*, et al.* A molecular mechanism for the topographic alignment of convergent neural maps. Elife 2017, 6. 10.7554/eLife.20470 PMC5360444 28322188

[24] Zhang Z, Liu WY, Diao YP, Xu W, Zhong YH, Zhang JY (2019). Superior colliculus GABAergic neurons are essential for acute dark induction of wakefulness in mice. Curr Biol.

[25] Sooksawate T, Yanagawa Y, Isa T (2012). Cholinergic responses in GABAergic and non-GABAergic neurons in the intermediate gray layer of mouse superior colliculus. Eur J Neurosci.

[26] Cebrian C, Parent A, Prensa L (2005). Patterns of axonal branching of neurons of the substantia nigra pars reticulata and pars lateralis in the rat. J Comp Neurol.

[27] Born G, Schmidt M (2004). Inhibition of superior colliculus neurons by a GABAergic input from the pretectal nuclear complex in the rat. Eur J Neurosci.

[28] Zhou Z, Liu X, Chen S, Zhang Z, Liu Y, Montardy Q (2019). A VTA GABAergic neural circuit mediates visually evoked innate defensive responses. Neuron.

[29] Shang C, Liu A, Li D, Xie Z, Chen Z, Huang M (2019). A subcortical excitatory circuit for sensory-triggered predatory hunting in mice. Nat Neurosci.

[30] Wang N, Perkins E, Zhou L, Warren S, May PJ (2013). Anatomical evidence that the superior colliculus controls saccades through central mesencephalic reticular formation gating of omnipause neuron activity. J Neurosci.

[31] Wei P, Liu N, Zhang Z, Liu X, Tang Y, He X (2015). Processing of visually evoked innate fear by a non-canonical thalamic pathway. Nat Commun.

[32] Evans DA, Stempel AV, Vale R, Ruehle S, Lefler Y, Branco T (2018). A synaptic threshold mechanism for computing escape decisions. Nature.

[33] Seabrook TA, Burbridge TJ, Crair MC, Huberman AD (2017). Architecture, function, and assembly of the mouse visual system. Annu Rev Neurosci.

[34] Gandhi NJ, Katnani HA (2011). Motor functions of the superior colliculus. Annu Rev Neurosci.

[35] Basso MA, Bickford ME, Cang J (2021). Unraveling circuits of visual perception and cognition through the superior colliculus. Neuron.

[36] Jun EJ, Bautista AR, Nunez MD, Allen DC, Tak JH, Alvarez E (2021). Causal role for the primate superior colliculus in the computation of evidence for perceptual decisions. Nat Neurosci.

[37] Krauzlis RJ, Lovejoy LP, Zénon A (2013). Superior colliculus and visual spatial attention. Annu Rev Neurosci.

[38] Wurtz RH, Goldberg ME (1972). The primate superior colliculus and the shift of visual attention. Invest Ophthalmol.

[39] Wang L, McAlonan K, Goldstein S, Gerfen CR, Krauzlis RJ (2020). A Causal role for mouse superior colliculus in visual perceptual decision-making. J Neurosci.

[40] Massot C, Jagadisan UK, Gandhi NJ (2019). Sensorimotor transformation elicits systematic patterns of activity along the dorsoventral extent of the superior colliculus in the macaque monkey. Commun Biol.

[41] Hikosaka O (2007). Basal ganglia mechanisms of reward-oriented eye movement. Ann N Y Acad Sci.

[42] Kaneda K, Isa K, Yanagawa Y, Isa T (2008). Nigral inhibition of GABAergic neurons in mouse superior colliculus. J Neurosci Off J Soc Neurosci.

[43] Herman JP, Arcizet F, Krauzlis RJ. Attention-related modulation of caudate neurons depends on superior colliculus activity. Elife 2020, 9. 10.7554/eLife.53998 PMC7544506 32940607

[44] Shang C, Chen Z, Liu A, Li Y, Zhang J, Qu B (2018). Divergent midbrain circuits orchestrate escape and freezing responses to looming stimuli in mice. Nat Commun.

[45] Shang C, Liu Z, Chen Z, Shi Y, Wang Q, Liu S*, et al.* BRAIN CIRCUITS. A parvalbumin-positive excitatory visual pathway to trigger fear responses in mice. Science 2015, 348: 1472-1477. 10.1126/science.aaa8694 26113723

[46] Zingg B, Chou XL, Zhang ZG, Mesik L, Liang F, Tao HW (2017). AAV-mediated anterograde transsynaptic tagging: mapping corticocollicular input-defined neural pathways for defense behaviors. Neuron.

[47] Favaro PD, Gouvea TS, de Oliveira SR, Vautrelle N, Redgrave P, Comoli E (2011). The influence of vibrissal somatosensory processing in rat superior colliculus on prey capture. Neuroscience.

[48] Zhang X, van den Pol AN. Rapid binge-like eating and body weight gain driven by zona incerta GABA neuron activation. Science (New York, N.Y.) 2017, 356: 853-859. 10.1126/science.aam7100 PMC6602535 28546212

[49] Cohen JD, Castro-Alamancos MA (2010). Behavioral state dependency of neural activity and sensory (whisker) responses in superior colliculus. J Neurophysiol.

[50] Rossi MA, Li HE, Lu D, Kim IH, Bartholomew RA, Gaidis E (2016). A GABAergic nigrotectal pathway for coordination of drinking behavior. Nat Neurosci.

[51] Villalobos CA, Wu Q, Lee PH, May PJ, Basso MA (2018). Parvalbumin and GABA Microcircuits in the Mouse Superior Colliculus. Front Neural Circ.

[52] Coimbra NC, De Oliveira R, Freitas RL, Ribeiro SJ, Borelli KG, Pacagnella RC (2006). Neuroanatomical approaches of the tectum-reticular pathways and immunohistochemical evidence for serotonin-positive perikarya on neuronal substrates of the superior colliculus and periaqueductal gray matter involved in the elaboration of the defensive behavior and fear-induced analgesia. Exp Neurol.

[53] Mooney RD, Huang X, Shi MY, Bennett-Clarke CA, Rhoades RW (1996). Serotonin modulates retinotectal and corticotectal convergence in the superior colliculus. Prog Brain Res.

[54] Binns KE, Salt TE (1997). Different roles for GABAA and GABAB receptors in visual processing in the rat superior colliculus. J Physiol.

[55] Endo T, Yanagawa Y, Obata K, Isa T (2003). Characteristics of GABAergic neurons in the superficial superior colliculus in mice. Neurosci Lett.

[56] Phongphanphanee P, Mizuno F, Lee PH, Yanagawa Y, Isa T, Hall WC (2011). A circuit model for saccadic suppression in the superior colliculus. J Neurosci.

[57] Dussaillant M, Sarrieau A, Gozes I, Berod A, Rostene W (1992). Distribution of cells expressing vasoactive intestinal peptide/peptide histidine isoleucine-amide precursor messenger RNA in the rat brain. Neuroscience.

[58] Harvey AR, Heavens RP, Yellachich LA, Sirinathsinghji DJ (2001). Expression of messenger RNAs for glutamic acid decarboxylase, preprotachykinin, cholecystokinin, somatostatin, proenkephalin and neuropeptide Y in the adult rat superior colliculus. Neuroscience.

[59] Ghitani N, Bayguinov PO, Vokoun CR, McMahon S, Jackson MB, Basso MA (2014). Excitatory synaptic feedback from the motor layer to the sensory layers of the superior colliculus. J Neurosci Off J Soc Neurosci.

[60] Pettit DL, Helms MC, Lee P, Augustine GJ, Hall WC (1999). Local excitatory circuits in the intermediate gray layer of the superior colliculus. J Neurophysiol.

[61] Olivier E, Corvisier J, Pauluis Q, Hardy O (2000). Evidence for glutamatergic tectotectal neurons in the cat superior colliculus: a comparison with GABAergic tectotectal neurons. Eur J Neurosci.

[62] Byun H, Kwon S, Ahn H-J, Liu H, Forrest D, Demb JB (2016). Molecular features distinguish ten neuronal types in the mouse superficial superior colliculus. J Comp Neurol.

[63] Masullo L, Mariotti L, Alexandre N, Freire-Pritchett P, Boulanger J, Tripodi M (2019). Genetically defined functional modules for spatial orienting in the mouse superior colliculus. Curr Biol CB.

[64] Hernandes MS, Lima LS, Scavone C, Lopes LR, Britto LR (2012). Eye enucleation activates the transcription nuclear factor kappa-B in the rat superior colliculus. Neurosci Lett.

[65] Thompson J, Lovicu F, Ziman M (2004). The role of Pax7 in determining the cytoarchitecture of the superior colliculus. Dev Growth Differ.

[66] Alvarez-Bolado G, Cecconi F, Wehr R, Gruss P (1999). The fork head transcription factor Fkh5/Mf3 is a developmental marker gene for superior colliculus layers and derivatives of the hindbrain somatic afferent zone. Brain Res Dev Brain Res.

[67] Buhusi M, Demyanenko GP, Jannie KM, Dalal J, Darnell EP, Weiner JA (2009). ALCAM regulates mediolateral retinotopic mapping in the superior colliculus. J Neurosci.

[68] Zhao J, Urakawa S, Matsumoto J, Li R, Ishii Y, Sasahara M (2013). Changes in Otx2 and parvalbumin immunoreactivity in the superior colliculus in the platelet-derived growth factor receptor-β knockout mice. BioMed Res Int.

[69] Li L, Feng X, Zhou Z, Zhang H, Shi Q, Lei Z (2018). Stress accelerates defensive responses to looming in mice and involves a locus coeruleus-superior colliculus projection. Curr Biol.

[70] Bolton AD, Murata Y, Kirchner R, Kim SY, Young A, Dang T (2015). A diencephalic dopamine source provides input to the superior colliculus, where D1 and D2 receptors segregate to distinct functional zones. Cell Rep.

[71] Baldwin MKL, Krubitzer L (2018). Architectonic characteristics of the visual thalamus and superior colliculus in titi monkeys. J Comp Neurol.

[72] da Silva JA, Almada RC, de Figueiredo RM, Coimbra NC (2018). Blockade of synaptic activity in the neostriatum and activation of striatal efferent pathways produce opposite effects on panic attack-like defensive behaviours evoked by GABAergic disinhibition in the deep layers of the superior colliculus. Physiol Behav.

[73] Lee PH, Sooksawate T, Yanagawa Y, Isa K, Isa T, Hall WC (2007). Identity of a pathway for saccadic suppression. Proc Natl Acad Sci U S A.

[74] Kaneda K, Phongphanphanee P, Katoh T, Isa K, Yanagawa Y, Obata K (2008). Regulation of burst activity through presynaptic and postsynaptic GABA(B) receptors in mouse superior colliculus. J Neurosci.

[75] Grabert J, Jost B, Patz S, Wahle P (2009). GABA(C) receptors are expressed in GABAergic and non-GABAergic neurons of the rat superior colliculus and visual cortex. Exp Brain Res.

[76] Binns KE (1999). The synaptic pharmacology underlying sensory processing in the superior colliculus. Prog Neurobiol.

[77] Cirone J, Sharp C, Jeffery G, Salt TE (2002). Distribution of metabotropic glutamate receptors in the superior colliculus of the adult rat, ferret and cat. Neuroscience.

[78] Islam R, Prater CM, Harris BN, Carr JA (2019). Neuroendocrine modulation of predator avoidance/prey capture tradeoffs: Role of tectal NPY2R receptors. General Comp Endocrinol.

[79] Boudin H, Pélaprat D, Rostène W, Beaudet A (1996). Cellular distribution of neurotensin receptors in rat brain: immunohistochemical study using an antipeptide antibody against the cloned high affinity receptor. J Comp Neurol.

[80] Liu X, Li X, Zhao G, Wang F, Wang L. Sexual dimorphic distribution of cannabinoid 1 receptor mRNA in adult C57BL/6J mice. J Comp Neurol 2020, 528: 1986–1999. 10.1002/cne.24868 31997354

[81] Ortiz C, Navarro JF, Jurek A, Märtin A, Lundeberg J, Meletis K. Molecular atlas of the adult mouse brain. Sci Adv 2020, 6: eabb3446. 10.1126/sciadv.abb3446 PMC7319762 32637622

[82] Chen WT, Lu A, Craessaerts K, Pavie B, Sala Frigerio C, Corthout N (2020). Spatial Transcriptomics and In Situ Sequencing to Study Alzheimer's Disease. Cell.

[83] Zador AM, Dubnau J, Oyibo HK, Zhan H, Cao G, Peikon ID. Sequencing the connectome. PLoS Biol 2012, 10: e1001411. 10.1371/journal.pbio.1001411 PMC3479097 23109909

[84] Close JL, Long BR, Zeng H (2021). Spatially resolved transcriptomics in neuroscience. Nat Methods.

[85] Qu J, Zhou X, Zhu H, Cheng G, Ashwell KW, Lu F (2006). Development of the human superior colliculus and the retinocollicular projection. Exp Eye Res.

[86] Perry VH, Cowey A (1984). Retinal ganglion cells that project to the superior colliculus and pretectum in the macaque monkey. Neuroscience.

[87] Cerkevich CM, Lyon DC, Balaram P, Kaas JH (2014). Distribution of cortical neurons projecting to the superior colliculus in macaque monkeys. Eye Brain.

[88] Baldwin MK, Kaas JH (2012). Cortical projections to the superior colliculus in prosimian galagos (Otolemur garnetti). J Comp Neurol.

[89] Liang F, Xiong XR, Zingg B, Ji XY, Zhang LI, Tao HW (2015). Sensory cortical control of a visually induced arrest behavior* via* corticotectal projections. Neuron.

[90] Wang Q, Burkhalter A (2013). Stream-related preferences of inputs to the superior colliculus from areas of dorsal and ventral streams of mouse visual cortex. J Neurosci.

[91] Comoli E, Coizet V, Boyes J, Bolam JP, Canteras NS, Quirk RH (2003). A direct projection from superior colliculus to substantia nigra for detecting salient visual events. Nat Neurosci.

[92] Comoli E, Das Neves Favaro P, Vautrelle N, Leriche M, Overton PG, Redgrave P. Segregated anatomical input to sub-regions of the rodent superior colliculus associated with approach and defense. Front Neuroanat 2012, 6: 9. 10.3389/fnana.2012.00009 PMC3324116 22514521

[93] Beltramo R, Scanziani M (2019). A collicular visual cortex: Neocortical space for an ancient midbrain visual structure. Science.

[94] Baldwin MKL, Young NA, Matrov D, Kaas JH (2019). Cortical projections to the superior colliculus in grey squirrels (Sciurus carolinensis). Eur J Neurosci.

[95] Goldberg ME, Wurtz RH (1972). Activity of superior colliculus in behaving monkey. II. Effect of attention on neuronal responses. J Neurophysiol.

[96] Humphrey NK (1968). Responses to visual stimuli of units in the superior colliculus of rats and monkeys. Exp Neurol.

[97] Schiller PH, Koerner F (1971). Discharge characteristics of single units in superior colliculus of the alert rhesus monkey. J Neurophysiol.

[98] Cynader M, Berman N (1972). Receptive-field organization of monkey superior colliculus. J Neurophysiol.

[99] Ellis EM, Gauvain G, Sivyer B, Murphy GJ (2016). Shared and distinct retinal input to the mouse superior colliculus and dorsal lateral geniculate nucleus. J Neurophysiol.

[100] Matsumoto M, Inoue KI, Takada M (2018). Causal role of neural signals transmitted from the frontal eye field to the superior colliculus in saccade generation. Front Neural Circuits.

[101] Huerta MF, Kaas JH (1990). Supplementary eye field as defined by intracortical microstimulation: connections in macaques. J Comp Neurol.

[102] Fregosi M, Rouiller EM (2017). Ipsilateral corticotectal projections from the primary, premotor and supplementary motor cortical areas in adult macaque monkeys: a quantitative anterograde tracing study. Eur J Neurosci.

[103] Borra E, Gerbella M, Rozzi S, Tonelli S, Luppino G (2014). Projections to the superior colliculus from inferior parietal, ventral premotor, and ventrolateral prefrontal areas involved in controlling goal-directed hand actions in the macaque. Cereb Cortex.

[104] Lynch JC, Graybiel AM, Lobeck LJ (1985). The differential projection of two cytoarchitectonic subregions of the inferior parietal lobule of macaque upon the deep layers of the superior colliculus. J Comp Neurol.

[105] Distler C, Hoffmann KP (2015). Direct projections from the dorsal premotor cortex to the superior colliculus in the macaque (macaca mulatta). J Comp Neurol.

[106] Jayaraman A, Batton RR, Carpenter MB (1977). Nigrotectal projections in the monkey: an autoradiographic study. Brain Res.

[107] Baldwin MK, Wei H, Reed JL, Bickford ME, Petry HM, Kaas JH (2013). Cortical projections to the superior colliculus in tree shrews (Tupaia belangeri). J Comp Neurol.

[108] Butler BE, Chabot N, Lomber SG (2016). A quantitative comparison of the hemispheric, areal, and laminar origins of sensory and motor cortical projections to the superior colliculus of the cat. J Comp Neurol.

[109] Zurita H, Rock C, Perkins J, Apicella AJ (2018). a layer-specific corticofugal input to the mouse superior colliculus. Cereb Cortex.

[110] Gale SD, Murphy GJ (2014). Distinct representation and distribution of visual information by specific cell types in mouse superficial superior colliculus. J Neurosci.

[111] Gale SD, Murphy GJ (2016). Active dendritic properties and local inhibitory input enable selectivity for object motion in mouse superior colliculus neurons. J Neurosci.

[112] Zhao X, Liu M, Cang J (2014). Visual cortex modulates the magnitude but not the selectivity of looming-evoked responses in the superior colliculus of awake mice. Neuron.

[113] Inayat S, Barchini J, Chen H, Feng L, Liu X, Cang J (2015). Neurons in the most superficial lamina of the mouse superior colliculus are highly selective for stimulus direction. J Neurosci.

[114] Beitz AJ, Clements JR, Mullett MA, Ecklund LJ (1986). Differential origin of brainstem serotoninergic projections to the midbrain periaqueductal gray and superior colliculus of the rat. J Comp Neurol.

[115] Kaneda K, Isa K, Yanagawa Y, Isa T (2008). Nigral inhibition of GABAergic neurons in mouse superior colliculus. J Neurosci.

[116] Sakata Y, Endoh H, Matsushige T, Furuya S, Nakamura S (2013). Asphyxia induced by umbilical cord occlusion alters glutamatergic and GABAergic synaptic transmission in neurons of the superior colliculus in fetal rats. Int J Dev Neurosci.

[117] Boka K, Chomsung R, Li J, Bickford ME (2006). Comparison of the ultrastructure of cortical and retinal terminals in the rat superior colliculus. Anat Rec A Discov Mol Cell Evol Biol.

[118] Bednarova V, Grothe B, Myoga MH (2018). Complex and spatially segregated auditory inputs of the mouse superior colliculus. J Physiol.

[119] Zhaoping L (2016). From the optic tectum to the primary visual cortex: migration through evolution of the saliency map for exogenous attentional guidance. Curr Opin Neurobiol.

[120] Kusunoki T, Amemiya F (1983). Retinal projections in the hagfish Eptatretus burgeri. Brain Res.

[121] von Twickel A, Kowatschew D, Saltürk M, Schauer M, Robertson B, Korsching S (2019). Individual dopaminergic neurons of lamprey SNc/VTA project to both the striatum and optic tectum but restrict co-release of glutamate to striatum only. Curr Biol.

[122] Dunn TW, Gebhardt C, Naumann EA, Riegler C, Ahrens MB, Engert F (2016). Neural circuits underlying visually evoked escapes in Larval Zebrafish. Neuron.

[123] Heap LAL, Vanwalleghem G, Thompson AW, Favre-Bulle IA, Scott EK (2018). Luminance changes drive directional startle through a thalamic pathway. Neuron.

[124] Mueller T (2012). What is the Thalamus in Zebrafish?. Front Neurosci.

[125] Heap LA, Vanwalleghem GC, Thompson AW, Favre-Bulle I, Rubinsztein-Dunlop H, Scott EK (2017). Hypothalamic projections to the optic tectum in Larval Zebrafish. Front Neuroanat.

[126] Henriques PM, Rahman N, Jackson SE, Bianco IH (2019). Nucleus Isthmi Is Required to Sustain Target Pursuit during Visually Guided Prey-Catching. Curr Biol.

[127] Gerits A, Farivar R, Rosen BR, Wald LL, Boyden ES, Vanduffel W (2012). Optogenetically induced behavioral and functional network changes in primates. Curr Biol.

[128] Poldrack RA, Farah MJ (2015). Progress and challenges in probing the human brain. Nature.

[129] Rafal RD, Koller K, Bultitude JH, Mullins P, Ward R, Mitchell AS (2015). Connectivity between the superior colliculus and the amygdala in humans and macaque monkeys: virtual dissection with probabilistic DTI tractography. J Neurophysiol.

[130] Kragel PA, Čeko M, Theriault J, Chen D, Satpute AB, Wald LW (2021). A human colliculus-pulvinar-amygdala pathway encodes negative emotion. Neuron.

[131] Baizer JS, Whitney JF, Bender DB (1991). Bilateral projections from the parabigeminal nucleus to the superior colliculus in monkey. Exp Brain Res.

[132] Harting JK (1977). Descending pathways from the superior collicullus: an autoradiographic analysis in the rhesus monkey (Macaca mulatta). J Comp Neurol.

[133] Baldwin MK, Balaram P, Kaas JH (2013). Projections of the superior colliculus to the pulvinar in prosimian galagos (Otolemur garnettii) and VGLUT2 staining of the visual pulvinar. J Comp Neurol.

[134] Sommer MA, Wurtz RH (2004). What the brain stem tells the frontal cortex. II. Role of the SC-MD-FEF pathway in corollary discharge. J Neurophysiol.

[135] Stepniewska I, Qi HX, Kaas JH (1999). Do superior colliculus projection zones in the inferior pulvinar project to MT in primates?. Eur J Neurosci.

[136] Zeater N, Buzás P, Dreher B, Grünert U, Martin PR (2019). Projections of three subcortical visual centers to marmoset lateral geniculate nucleus. J Comp Neurol.

[137] Feig S, Van Lieshout DP, Harting JK (1992). Ultrastructural studies of retinal, visual cortical (area 17), and parabigeminal terminals within the superior colliculus of Galago crassicaudatus. J Comp Neurol.

[138] Graybiel AM (1978). A satellite system of the superior colliculus: the parabigeminal nucleus and its projections to the superficial collicular layers. Brain Res.

[139] Fernandes AM, Mearns DS, Donovan JC, Larsch J, Helmbrecht TO, Kölsch Y (2021). Neural circuitry for stimulus selection in the zebrafish visual system. Neuron.

[140] Isa T, Marquez-Legorreta E, Grillner S, Scott EK (2021). The tectum/superior colliculus as the vertebrate solution for spatial sensory integration and action. Curr Biol.

[141] Kolmac CI, Mitrofanis J (1998). Patterns of brainstem projection to the thalamic reticular nucleus. J Comp Neurol.

[142] Furigo IC, de Oliveira WF, de Oliveira AR, Comoli E, Baldo MV, Mota-Ortiz SR (2010). The role of the superior colliculus in predatory hunting. Neuroscience.

[143] Coizet V, Graham JH, Moss J, Bolam JP, Savasta M, McHaffie JG (2009). Short-latency visual input to the subthalamic nucleus is provided by the midbrain superior colliculus. J Neurosci.

[144] Tokuno H, Takada M, Ikai Y, Mizuno N (1994). Direct projections from the deep layers of the superior colliculus to the subthalamic nucleus in the rat. Brain Res.

[145] McKenna JT, Vertes RP (2004). Afferent projections to nucleus reuniens of the thalamus. J Comp Neurol.

[146] Kaneshige M, Shibata KI, Matsubayashi J, Mitani A, Furuta T (2018). A descending circuit derived from the superior colliculus modulates vibrissal movements. Front Neural Circuits.

[147] Redgrave P, Dean P, Mitchell IJ, Odekunle A, Clark A (1988). The projection from superior colliculus to cuneiform area in the rat I. Anatomical studies. Exp Brain Res.

[148] Watson GD, Smith JB, Alloway KD (2015). The zona incerta regulates communication between the superior colliculus and the posteromedial thalamus: implications for thalamic interactions with the dorsolateral striatum. J Neurosci.

[149] Son S, Manjila SB, Newmaster KT, Wu Y-t, Vanselow DJ, Ciarletta M*, et al.* Wiring diagram of the oxytocin system in the mouse brain. bioRxiv 2020: 2020.2010.2001.320978.

[150] Wang X, Yang H, Pan L, Hao S, Wu X, Zhan L (2019). Brain-wide mapping of mono-synaptic afferents to different cell types in the laterodorsal tegmentum. Neurosci Bull.

[151] Baek J, Lee S, Cho T, Kim SW, Kim M, Yoon Y (2019). Neural circuits underlying a psychotherapeutic regimen for fear disorders. Nature.

[152] Helmbrecht TO, Dal Maschio M, Donovan JC, Koutsouli S, Baier H (2018). Topography of a Visuomotor Transformation. Neuron.

[153] Tardif E, Clarke S (2002). Commissural connections of human superior colliculus. Neuroscience.

[154] Edwards SB (1977). The commissural projection of the superior colliculus in the cat. J Comp Neurol.

[155] Watanabe K, Kawana E (1979). Efferent projections of the parabigeminal nucleus in rats: a horseradish peroxidase (HRP) study. Brain Res.

[156] Sahibzada N, Yamasaki D, Rhoades RW (1987). The spinal and commissural projections from the superior colliculus in rat and hamster arise from distinct neuronal populations. Brain Res.

[157] Robert F, Cuénod M (1969). Electrophysiology of the intertectal commissures in the pigeon. II. Inhibitory interaction. Exp Brain Res.

[158] Hoffmann KP, Straschill M (1971). Influences of cortico-tectal and intertectal connections on visual responses in the cat's superior colliculus. Exp Brain Res.

[159] Goodale MA (1973). Cortico-tectal and intertectal modulation of visual responses in the rat's superior colliculus. Exp Brain Res.

[160] Wurtz RH, Mohler CW (1976). Organization of monkey superior colliculus: enhanced visual response of superficial layer cells. J Neurophysiol.

[161] Tardif E, Delacuisine B, Probst A, Clarke S (2005). Intrinsic connectivity of human superior colliculus. Exp Brain Res.

[162] Isa T, Endo T, Saito Y (1998). The visuo-motor pathway in the local circuit of the rat superior colliculus. J Neurosci Off J Soc Neurosci.

[163] Lanyon LJ, Giaschi D, Young SA, Fitzpatrick K, Diao L, Bjornson BH (2009). Combined functional MRI and diffusion tensor imaging analysis of visual motion pathways. J Neuroophthalmol.

[164] Olivé I, Densmore M, Harricharan S, Théberge J, McKinnon MC, Lanius R (2018). Superior colliculus resting state networks in post-traumatic stress disorder and its dissociative subtype. Hum Brain Mapp.

[165] Bogadhi AR, Katz LN, Bollimunta A, Leopold DA, Krauzlis RJ (2021). Midbrain activity shapes high-level visual properties in the primate temporal cortex. Neuron.

[166] Olivé I, Tempelmann C, Berthoz A, Heinze HJ (2015). Increased functional connectivity between superior colliculus and brain regions implicated in bodily self-consciousness during the rubber hand illusion. Hum Brain Mapp.

[167] May PJ, Basso MA (2018). Connections between the zona incerta and superior colliculus in the monkey and squirrel. Brain Struct Funct.

[168] Sans-Dublanc A, Chrzanowska A, Reinhard K, Lemmon D, Nuttin B, Lambert T (2021). Optogenetic fUSI for brain-wide mapping of neural activity mediating collicular-dependent behaviors. Neuron.

[169] Wurtz RH, Goldberg ME (1971). Superior colliculus cell responses related to eye movements in awake monkeys. Science.

[170] Wurtz RH, Optican LM (1994). Superior colliculus cell types and models of saccade generation. Curr Opin Neurobiol.

[171] Sparks DL (1986). Translation of sensory signals into commands for control of saccadic eye movements: role of primate superior colliculus. Physiol Rev.

[172] Robinson DA (1972). Eye movements evoked by collicular stimulation in the alert monkey. Vision Res.

[173] Mohler CW, Wurtz RH (1976). Organization of monkey superior colliculus: intermediate layer cells discharging before eye movements. J Neurophysiol.

[174] Hayashi Y, Nagata T, Shiomi K (1977). Electrical activity of superior colliculus associated with eye movements in alert cats. Tohoku J Exp Med.

[175] May PJ, Sun W, Hall WC (1997). Reciprocal connections between the zona incerta and the pretectum and superior colliculus of the cat. Neuroscience.

[176] McHaffie JG, Stein BE (1982). Eye movements evoked by electrical stimulation in the superior colliculus of rats and hamsters. Brain Res.

[177] Wang N, Perkins E, Zhou L, Warren S, May PJ (2017). Reticular formation connections underlying horizontal gaze: the central mesencephalic reticular formation (cMRF) as a conduit for the collicular saccade signal. Frontiers in neuroanatomy.

[178] Fang Q, Chou X-L, Peng B, Zhong W, Zhang LI, Tao HW (2020). A differential circuit via retino-colliculo-pulvinar pathway enhances feature selectivity in visual cortex through surround suppression. Neuron.

[179] James, William. The principles of psychology, Vol I. 1890, 10.1037/10538-000.

[180] Kastner S, Ungerleider LG (2000). Mechanisms of visual attention in the human cortex. Annu Rev Neurosci.

[181] Battistoni E, Stein T, Peelen MV (2017). Preparatory attention in visual cortex. Ann N Y Acad Sci.

[182] Behrmann M, Geng JJ, Shomstein S (2004). Parietal cortex and attention. Curr Opin Neurobiol.

[183] Fiebelkorn IC, Kastner S (2020). Functional Specialization in the Attention Network. Annu Rev Psychol.

[184] Basso MA, May PJ (2017). Circuits for action and cognition: a view from the superior colliculus. Annu Rev Vis Sci.

[185] Lovejoy LP, Krauzlis RJ (2010). Inactivation of primate superior colliculus impairs covert selection of signals for perceptual judgments. Nat Neurosci.

[186] Hu F, Dan Y. An inferior-superior colliculus circuit controls auditory cue-directed visual spatial attention. Neuron 2022, 110: 109–119. 10.1016/j.neuron.2021.10.004 34699777

[187] Kojima J, Matsumura M, Togawa M, Hikosaka O (1996). Tonic activity during visuo-oculomotor behavior in the monkey superior colliculus. Neurosci Res.

[188] Müller JR, Philiastides MG, Newsome WT (2005). Microstimulation of the superior colliculus focuses attention without moving the eyes. Proc Natl Acad Sci U S A.

[189] Cavanaugh J, Alvarez BD, Wurtz RH (2006). Enhanced performance with brain stimulation: attentional shift or visual cue?. J Neurosci.

[190] Cavanaugh J, Wurtz RH (2004). Subcortical modulation of attention counters change blindness. J Neurosci.

[191] Glimcher PW, Sparks DL (1992). Movement selection in advance of action in the superior colliculus. Nature.

[192] Basso MA, Wurtz RH (1998). Modulation of neuronal activity in superior colliculus by changes in target probability. J Neurosci.

[193] Basso MA, Wurtz RH (1997). Modulation of neuronal activity by target uncertainty. Nature.

[194] McPeek RM, Keller EL (2004). Deficits in saccade target selection after inactivation of superior colliculus. Nat Neurosci.

[195] Nummela SU, Krauzlis RJ (2010). Inactivation of primate superior colliculus biases target choice for smooth pursuit, saccades, and button press responses. J Neurophysiol.

[196] Song JH, Rafal RD, McPeek RM (2011). Deficits in reach target selection during inactivation of the midbrain superior colliculus. Proc Natl Acad Sci U S A.

[197] Felsen G, Mainen ZF (2008). Neural substrates of sensory-guided locomotor decisions in the rat superior colliculus. Neuron.

[198] Crapse TB, Lau H, Basso MA (2018). A Role for the Superior Colliculus in Decision Criteria. Neuron.

[199] Yilmaz M, Meister M (2013). Rapid innate defensive responses of mice to looming visual stimuli. Curr Biol.

[200] Billington J, Wilkie RM, Field DT, Wann JP (2011). Neural processing of imminent collision in humans. Proc Biol Sci.

[201] King SM, Cowey A (1992). Defensive responses to looming visual stimuli in monkeys with unilateral striate cortex ablation. Neuropsychologia.

[202] Liu YJ, Wang Q, Li B (2011). Neuronal responses to looming objects in the superior colliculus of the cat. Brain Behav Evol.

[203] Tischler MD, Davis M (1983). A visual pathway that mediates fear-conditioned enhancement of acoustic startle. Brain Res.

[204] Bhattacharyya K, McLean DL, MacIver MA (2017). Visual threat assessment and reticulospinal encoding of calibrated responses in Larval Zebrafish. Curr Biol.

[205] Duggan O, Narasimham S, Govern EM, Killian O, O'Riordan S, Hutchinson M (2019). A study of the midbrain network for covert attentional orienting in cervical dystonia patients using dynamic causal modelling. Conf Proc IEEE Eng Med Biol Soc.

[206] Wu LQ, Niu YQ, Yang J, Wang SR (2005). Tectal neurons signal impending collision of looming objects in the pigeon. Eur J Neurosci.

[207] Khakhalin AS, Koren D, Gu J, Xu H, Aizenman CD (2014). Excitation and inhibition in recurrent networks mediate collision avoidance in Xenopus tadpoles. Eur J Neurosci.

[208] Nakagawa H, Hongjian K (2010). Collision-sensitive neurons in the optic tectum of the bullfrog. Rana catesbeiana. J Neurophysiol.

[209] Temizer I, Donovan JC, Baier H, Semmelhack JL (2015). A Visual pathway for looming-evoked escape in larval zebrafish. Curr Biol.

[210] Yin C, Li X, Du J (2019). Optic tectal superficial interneurons detect motion in larval zebrafish. Protein Cell.

[211] Bollmann JH (2019). The Zebrafish Visual System: From Circuits to Behavior. Annu Rev Vis Sci.

[212] Förster D, Helmbrecht TO, Mearns DS, Jordan L, Mokayes N, Baier H. Retinotectal circuitry of larval zebrafish is adapted to detection and pursuit of prey. Elife 2020, 9. 10.7554/eLife.58596 PMC7550190 33044168

[213] Gauvain G, Murphy GJ (2015). Projection-specific characteristics of retinal input to the brain. J Neurosci.

[214] Barker AJ, Helmbrecht TO, Grob AA, Baier H (2021). Functional, molecular and morphological heterogeneity of superficial interneurons in the larval zebrafish tectum. J Comp Neurol.

[215] Perez-Fernandez J, Kardamakis AA, Suzuki DG, Robertson B, Grillner S. Direct dopaminergic projections from the SNc modulate visuomotor transformation in the lamprey tectum. Neuron 2017, 96: 910–924 e915. 10.1016/j.neuron.2017.09.051 29107519

[216] Zhao ZD, Chen Z, Xiang X, Hu M, Xie H, Jia X (2019). Zona incerta GABAergic neurons integrate prey-related sensory signals and induce an appetitive drive to promote hunting. Nat Neurosci.

[217] Hoy JL, Bishop HI, Niell CM. Defined cell types in superior colliculus make distinct contributions to prey capture behavior in the mouse. Curr Biol 2019, 29: 4130–4138 e4135. 10.1016/j.cub.2019.10.017 PMC6925587 31761701

[218] Miller AM, Obermeyer WH, Behan M, Benca RM (1998). The superior colliculus-pretectum mediates the direct effects of light on sleep. Proc Natl Acad Sci U S A.

[219] Huang M, Li D, Cheng X, Pei Q, Xie Z, Gu H (2021). The tectonigral pathway regulates appetitive locomotion in predatory hunting in mice. Nat Commun.

[220] Almada RC, Genewsky AJ, Heinz DE, Kaplick PM, Coimbra NC, Wotjak CT (2018). Stimulation of the nigrotectal pathway at the level of the superior colliculus reduces threat recognition and causes a shift from avoidance to approach behavior. Front Neural Circuits.

[221] Barker AJ, Baier H (2015). Sensorimotor decision making in the zebrafish tectum. Current biology : CB.

[222] Filosa A, Barker AJ, Dal Maschio M, Baier H (2016). Feeding state modulates behavioral choice and processing of prey stimuli in the Zebrafish Tectum. Neuron.

[223] Del Bene F, Wyart C, Robles E, Tran A, Looger L, Scott EK (2010). Filtering of visual information in the tectum by an identified neural circuit. Science (New York, NY).

[224] Siegel JM (2009). Sleep viewed as a state of adaptive inactivity. Nature Reviews Neuroscience.

[225] Lupi D, Oster H, Thompson S, Foster RG (2008). The acute light-induction of sleep is mediated by OPN4-based photoreception. Nature neuroscience.

[226] Li Y, Lopez-Huerta VG, Adiconis X, Levandowski K, Choi S, Simmons SK (2020). Distinct subnetworks of the thalamic reticular nucleus. Nature.

[227] Fowler SC, Mortell C (1992). Low doses of haloperidol interfere with rat tongue extensions during licking: a quantitative analysis. Behavioral neuroscience.

[228] Taha EB, Dean P, Redgrave P (1982). Oral behaviour induced by intranigral muscimol is unaffected by haloperidol but abolished by large lesions of superior colliculus. Psychopharmacology.

[229] Villalobos CA, Basso MA. Optogenetic activation of the inhibitory nigro-collicular circuit evokes orienting movements in mice. bioRxiv 2020: 2020.2005.2021.107680. 10.1016/j.celrep.2022.110699 PMC10144672 35443172

[230] McFadyen J, Dolan RJ, Garrido MI (2020). The influence of subcortical shortcuts on disordered sensory and cognitive processing. Nat Rev Neurosci.

[231] Hu Y, Chen Z, Huang L, Xi Y, Li B, Wang H (2017). A translational study on looming-evoked defensive response and the underlying subcortical pathway in autism. Sci Rep.

[232] Kay RB, Gabreski NA, Triplett JW (2018). Visual subcircuit-specific dysfunction and input-specific mispatterning in the superior colliculus of fragile X mice. J Neurodev Disord.

[233] Pretegiani E, Vanegas-Arroyave N, FitzGibbon EJ, Hallett M, Optican LM (2019). Evidence from Parkinson's disease that the superior colliculus couples action and perception. Mov Disord.

[234] Cubizolle S, Damon-Perrière N, Dupouy S, Foubert-Samier A, Tison F (2014). Parkinson's disease, L-Dopa and "express" saccades: superior colliculus dyskinesias?. Clin Neurophysiol.

[235] Rolland M, Carcenac C, Overton PG, Savasta M, Coizet V (2013). Enhanced visual responses in the superior colliculus and subthalamic nucleus in an animal model of Parkinson's disease. Neuroscience.

[236] Erskine D, Thomas AJ, Taylor JP, Savage MA, Attems J, McKeith IG (2017). Neuronal loss and Α-synuclein pathology in the superior colliculus and its relationship to visual hallucinations in dementia with lewy bodies. Am J Geriatr Psychiatry.

[237] Clements KM, Devonshire IM, Reynolds JN, Overton PG (2014). Enhanced visual responses in the superior colliculus in an animal model of attention-deficit hyperactivity disorder and their suppression by D-amphetamine. Neuroscience.

[238] Depaulis A, Liu Z, Vergnes M, Marescaux C, Micheletti G, Warter JM (1990). Suppression of spontaneous generalized non-convulsive seizures in the rat by microinjection of GABA antagonists into the superior colliculus. Epilepsy Res.

[239] Nail-Boucherie K, Lê-Pham BT, Marescaux C, Depaulis A (2002). Suppression of absence seizures by electrical and pharmacological activation of the caudal superior colliculus in a genetic model of absence epilepsy in the rat. Exp Neurol.

[240] Redgrave P, Dean P, Simkins M (1988). Intratectal glutamate suppresses pentylenetetrazole-induced spike-and-wave discharges. Eur J Pharmacol.

[241] Soper C, Wicker E, Kulick CV, N'Gouemo P, Forcelli PA (2016). Optogenetic activation of superior colliculus neurons suppresses seizures originating in diverse brain networks. Neurobiol Dis.

[242] Revishchin AV, Solus GM, Poletaeva II, Pavlova GV (2018). Audiogenic epilepsy and structural features of superior colliculus in KM rats. Dokl Biochem Biophys.

[243] Diederich NJ, Stebbins G, Schiltz C, Goetz CG (2014). Are patients with Parkinson's disease blind to blindsight?. Brain.

[244] Nemanich ST, Earhart GM (2016). Freezing of gait is associated with increased saccade latency and variability in Parkinson's disease. Clin Neurophysiol.

[245] Pieruccini-Faria F, Jones JA, Almeida QJ (2014). Motor planning in Parkinson's disease patients experiencing freezing of gait: the influence of cognitive load when approaching obstacles. Brain Cogn.

[246] Soares SC, Maior RS, Isbell LA, Tomaz C, Nishijo H (2017). Fast detector/first responder: interactions between the superior colliculus-pulvinar pathway and stimuli relevant to primates. Front Neurosci.

[247] Basso MA, Powers AS, Evinger C (1996). An explanation for reflex blink hyperexcitability in Parkinson's disease. I. Superior colliculus. J Neurosci.

[248] Moro E, Bellot E, Meoni S, Pelissier P, Hera R, Dojat M (2020). Visual dysfunction of the superior colliculus in de novo parkinsonian patients. Ann Neurol.

[249] Dean P, Gale K (1989). Anticonvulsant action of GABA receptor blockade in the nigrotectal target region. Brain Res.

[250] Garant DS, Gale K (1987). Substantia nigra-mediated anticonvulsant actions: role of nigral output pathways. Exp Neurol.

[251] Wicker E, Beck VC, Kulick-Soper C, Kulick-Soper CV, Hyder SK, Campos-Rodriguez C (2019). Descending projections from the substantia nigra pars reticulata differentially control seizures. Proc Natl Acad Sci U S A.

[252] Ribak CE, Khurana V, Lien NT (1994). The effect of midbrain collicular knife cuts on audiogenic seizure severity in the genetically epilepsy-prone rat. J Hirnforsch.

[253] Ribak CE, Manio AL, Navetta MS, Gall CM (1997). In situ hybridization for c-fos mRNA reveals the involvement of the superior colliculus in the propagation of seizure activity in genetically epilepsy-prone rats. Epilepsy Res.

[254] Nail-Boucherie K, Le-Pham BT, Gobaille S, Maitre M, Aunis D, Depaulis A (2005). Evidence for a role of the parafascicular nucleus of the thalamus in the control of epileptic seizures by the superior colliculus. Epilepsia.

[255] Overton PG (2008). Collicular dysfunction in attention deficit hyperactivity disorder. Med Hypotheses.

